# Antigen targeting reveals splenic CD169^+^ macrophages as promoters of germinal center B‐cell responses

**DOI:** 10.1002/eji.201444983

**Published:** 2015-01-14

**Authors:** Henrike Veninga, Ellen G. F. Borg, Kyle Vreeman, Philip R. Taylor, Hakan Kalay, Yvette van Kooyk, Georg Kraal, Luisa Martinez‐Pomares, Joke M.M. den Haan

**Affiliations:** ^1^ Department of Molecular Cell Biology and ImmunologyVU University Medical CenterAmsterdamThe Netherlands; ^2^ Sir William Dunn School of PathologyUniversity of OxfordOxfordUK

**Keywords:** B‐cell response, CD169, Germinal center, Macrophage, Spleen

## Abstract

Ag delivery to specific APCs is an attractive approach in developing strategies for vaccination. CD169^+^ macrophages in the marginal zone of the spleen represent a suitable target for delivery of Ag because of their strategic location, which is optimal for the capture of blood‐borne Ag and their close proximity to B cells and T cells in the white pulp. Here we show that Ag targeting to CD169^+^ macrophages in mice resulted in strong, isotype‐switched, high‐affinity Ab production and the preferential induction and long‐term persistence of Ag‐specific GC B cells and follicular Th cells. In agreement with these observations, CD169^+^ macrophages retained intact Ag, induced cognate activation of B cells, and increased expression of costimulatory molecules upon activation. In addition, macrophages were required for the production of cytokines that promote B‐cell responses. Our results identify CD169^+^ macrophages as promoters of high‐affinity humoral immune responses and emphasize the value of CD169 as target for Ag delivery to improve vaccine responses.

## Introduction

Targeting Ag to APCs represents an attractive approach to improve vaccine efficiency 1, 2. mAbs recognizing surface molecules expressed on APCs have been successfully exploited to deliver Ag to specific APC subsets and thereby provide a tool to enhance specific immune responses and to control the cell type that presents the Ag to the immune system. Because of their potent capacity to capture, process, and present Ag to T cells, Ag‐targeting studies have focused on subsets of DCs and improved CTL responses against tumors 3, 4, 5, 6, 7, 8. More recently the induction of humoral immunity via Ag targeting to DC subsets has gained renewed interest 9, 10, 11, 12, 13, 14, 15, 16.

CD169^+^ macrophages are a subset of macrophages strategically located in the marginal zone of the spleen and the subcapsular sinus (SCS) of LNs at the entry site of blood or lymph fluid, respectively. A vast array of older and recent studies have indicated an important role for CD169^+^ macrophages in the capture of pathogens 19, 20, 21, 22, the early production of proinflammatory cytokines 23, 24, 25, 26, and the prevention of further dissemination of the infection 21, 25, 26.

CD169^+^ macrophages have also been shown to play a role in Ag presentation and induction of adaptive immune responses. SCS CD169^+^ macrophages in the LN activate iNKT cells in a CD1d‐dependent manner 27, 28 and capture and transfer immune complexes and viruses to B cells 29, 30, 31, 32. Furthermore, previous work from our lab showed that Ag targeted to splenic CD169^+^ macrophages is transferred to CD8^+^ DCs for cross‐presentation and thereby stimulates potent CD8^+^ T‐cell responses 33. Together these studies indicate that CD169^+^ macrophages are specialized in Ag uptake and transfer to other immune cells and suggest that these cells may prove to be attractive targeting candidates for new vaccination strategies.

Here we investigated humoral responses induced by Ag delivered to CD169^+^ macrophages using two targeting strategies and in comparison with Ag targeting to DEC205^+^ DCs. Ag targeting to CD169^+^ macrophages resulted in strong, high‐affinity, isotype‐switched Ab production and the induction and persistence of Ag‐specific GC B cells. This response was T cell dependent and induced efficient follicular Th (Tfh) cell differentiation. Interestingly, we detected activation of cognate B cells and prolonged retention of intact Ag on CD169^+^ macrophages. In addition, upon immunization CD169^+^ macrophages increased expression of costimulatory molecules and macrophages were required for the induction of cytokines and chemokines that promote B‐cell responses. Overall, this study shows that CD169^+^ macrophages are potent inducers of humoral immunity via the promotion of GC B‐cell responses. Together with our previous study demonstrating that Ag targeting to CD169^+^ macrophages results in strong CD8^+^ T‐cell responses, our findings strongly support an important role for CD169^+^ macrophages in the induction of both cellular and humoral immune responses and as suitable candidates for the development of new APC targeting based vaccination strategies.

## Results

### Ag targeting to CD169^+^ macrophages leads to strong Ab responses

**Figure 1:**
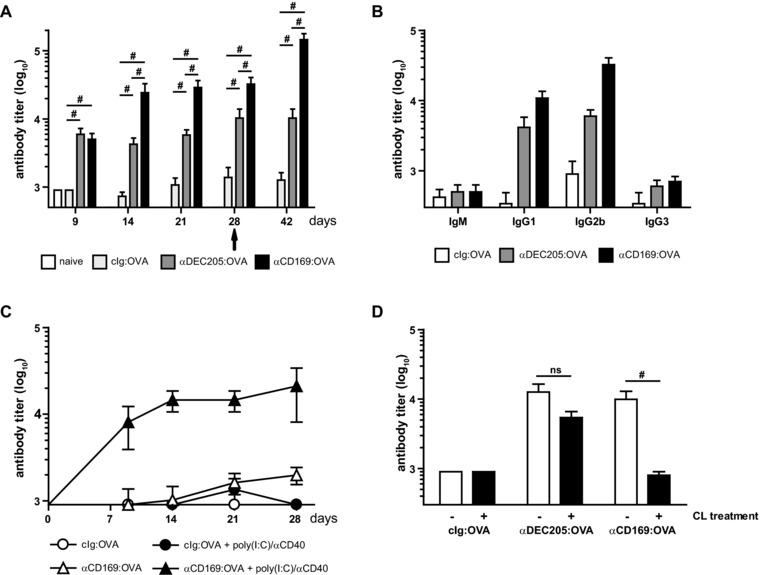
Ag targeting to CD169^+^ macrophages induces anti‐OVA Ab responses. (A) B6 mice were i.v. immunized with 1 μg mAb:OVA together with 25 μg αCD40 and 25 μg poly(I:C) and boosted at day 28 with 1 μg OVA:NP (indicated by arrow). OVA‐specific serum Abs were analyzed by ELISA at indicated time points. Serum dilution with OD450 > 0.1 is shown as mean ± SEM of one experiment representative of six (day 9), one (days 14, 21, 42), and three (day 28) independent experiments performed using four to seven mice/group/day in each experiment. (B) B6 mice were immunized i.v. with 1 μg mAb:OVA together with 25 μg αCD40 and 25 μg poly(I:C). Isotype‐specific anti‐OVA serum Abs were analyzed after 28 days by ELISA Graph shows the serum dilution (mean ± SEM) with OD450 > 0.1 of one representative experiment out of three independent experiments using five to seven mice/group per experiment. (C) B6 mice were injected i.v. with αCD169:OVA (triangles) or cIg:OVA (circles) in the absence (white symbols) or presence (black symbols) of 25 μg αCD40 and 25 μg poly(I:C). OVA‐specific serum Ab titers were analyzed on the indicated days by ELISA. Serum dilution with OD450 > 0.1 are shown as mean ± SEM from a single experiment using four to five mice/group/day. (D) B6 mice were immunized with 1 μg mAb:OVA together with 25 μg αCD40 and 25 μg poly(I:C). Mice were either untreated (white bars) or i.v. injected with clodronate liposomes (CLs, black bars) 7–8 days prior to immunization to deplete macrophages from the marginal zone. Anti‐OVA serum Abs were analyzed after 9 days by ELISA. Serum dilutions with OD450 > 0.1 are shown as mean ± SEM for one representative experiment from two independent experiments using six mice/group per experiment. (A, B, D) Data were analyzed by a Kruskal–Wallis test with Bonferroni's correction; *p*‐value indicator # refers to *p* < 0.0167, ns = not significant.

OVA was conjugated to mAbs specific for CD169 or DEC205 to target macrophages and DCs in the spleen, respectively. Control experiments confirmed the specific binding capacity of the conjugates to CD169^+^ macrophages and DEC205^+^ DCs (Supporting Information Fig. 1A and B) and that the conjugation efficiency was similar for all mAb:OVA conjugates (Supporting Information Fig. 1C). Targeting to CD169^+^ macrophages was superior in the induction of anti‐OVA Ab responses at days 14–28 after immunization and this was also reflected in a significantly higher recall response when the animals were boosted at day 28 with 1 μg free OVA (Fig. 1A).

Anti‐OVA Abs induced by anti(α)CD169:OVA consisted mostly of IgG1 and IgG2b isotypes with little IgM and IgG3 produced (Fig. 1B). The B‐cell response was dependent on the supplementation of adjuvant as targeting with αCD169:OVA without αCD40 and poly(I:C) hardly resulted in detectable levels of anti‐OVA Abs over time (Fig. 1C). Depletion of macrophages in the marginal zone using clodronate liposomes (CLs) 34 led to abrogation of the αCD169 but not αDEC205‐targeted responses, which illustrates the specificity of CD169^+^ macrophages targeting (Fig. 1D and Supporting Information Fig. 2). Together, these results indicate that Ag targeting to CD169^+^ macrophages in the presence of adjuvant results in the generation of isotype‐switched Ab responses.

### CD169^+^ macrophages preferentially enhance the GC pathway

**Figure 2:**
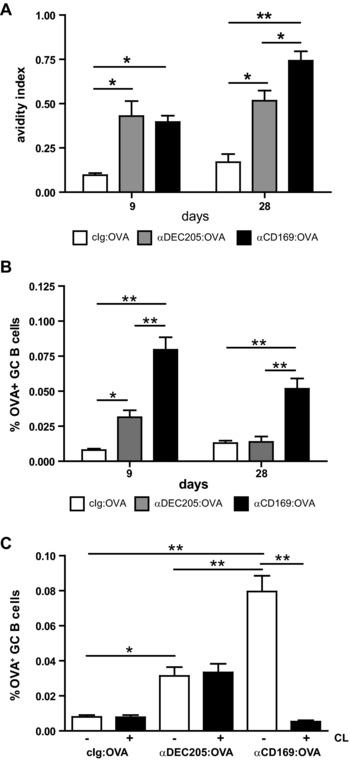
CD169^+^ macrophages preferentially enhance the GC pathway. (A) B6 mice were immunized i.v. with 1 μg mAb:OVA together with 25 μg αCD40 and 25 μg poly(I:C). OVA‐specific Abs in serum were detected by avidity ELISA at indicated time points; the avidity index is the concentration of NH_4_SCN needed for a 50% reduction of the OD450 in the absence of NH_4_SCN. Avidity indexes are shown as mean ± SEM from one representative experiment out of three independent experiments using five to seven mice/group in each experiment. (B) B6 mice were i.v. immunized with 1 μg mAb:OVA together with 25 μg αCD40 and 25 μg poly(I:C). OVA‐specific GC B cells in the spleen were analyzed by flow cytometry at indicated time points. Gating strategy is shown in Supporting Information Fig. 3. Gates are set on fluorescence minus one. OVA‐specific GC B cells were gated as live, single, non‐AF, B220^+^, CD38^−^, GL7^+^, OVA^+^ cells. Percentages of OVA‐specific GC B cells out of total B cells are shown as mean ± SEM from one representative experiment from four (day 9) or two (day 28) independent experiments using five to seven mice/group in each experiment. (C) B6 mice were i.v. immunized with 1 μg mAb:OVA together with 25 μg αCD40 and 25 μg poly(I:C). Mice were either untreated (white bars) or CL treated (black bars) 8 days prior to immunization. OVA‐specific GC B cells in the spleen were analyzed by flow cytometry after 9 days. OVA‐specific GC B cells as percentage of total B cells gated as described in (B) are shown as mean ± SEM of a single experiment using six mice/group. All data were analyzed by ANOVA with Bonferroni's correction; *p*‐value indicator * and ** refers to *p* < 0.05 and *p* < 0.005, respectively.

The presence of isotype‐switched Abs points to an active GC formation and affinity maturation. We therefore tested the overall avidity of the Ab response after CD169^+^ macrophages and DEC205^+^ DCs targeting at days 9 and 28 after immunization (Fig. 2A). CD169 targeting led to higher avidity Abs at day 28 after immunization, which was also reflected in the percentage of OVA‐specific GC B cells present in mice. Mice immunized with αCD169 conjugates showed considerable percentages of OVA‐specific GC B cells 28 days after immunization, whereas in DEC205‐targeted mice their numbers had already significantly declined at this time point (Fig. 2B, Supporting Information Fig. 3). Furthermore, the induction of OVA‐specific GC B cells was completely dependent on the presence of macrophages in the marginal zone (Fig. 2C). Overall, these data demonstrate that Ag targeting to splenic CD169^+^ macrophages leads to enhanced and possibly prolonged GC activity with higher affinity Abs compared to DEC205^+^ DC targeting.

### Tfh‐cell responses are essential for B‐cell responses after Ag targeting to CD169^+^ macrophages

**Figure 3:**
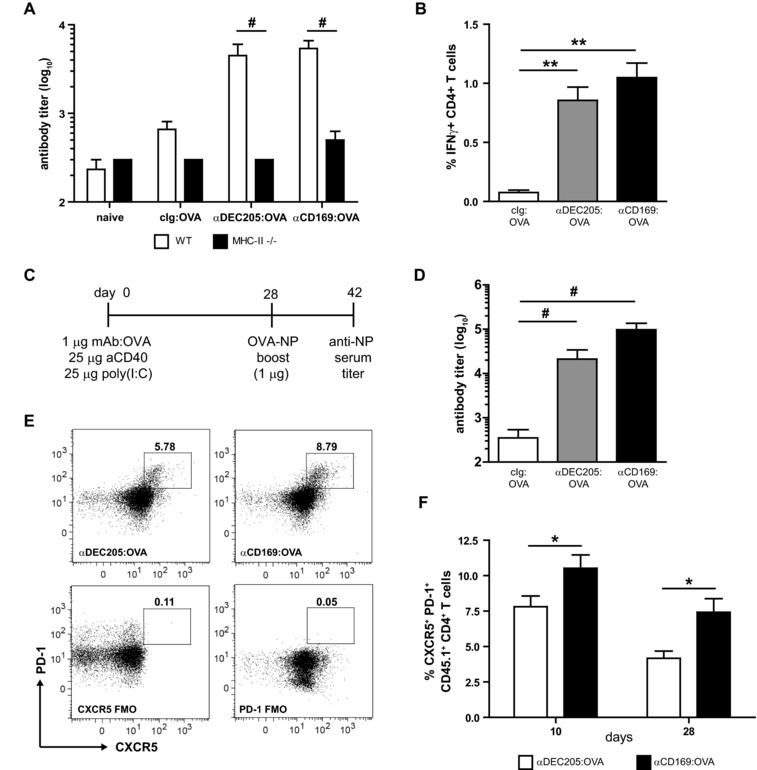
Tfh‐cell responses are essential for B‐cell responses after Ag targeting to CD169^+^ macrophages. (A) B6 (white bars) or MHC‐II^−/−^ B6 mice (black bars) were i.v. immunized with 1 μg of indicated mAb:OVA together with 25 μg αCD40 and 25 μg poly(I:C). OVA‐specific Ab titers in serum were analyzed by ELISA after 9 days. Serum dilutions with OD450 > 0.1 are shown as mean ± SEM from a single experiment with five to seven mice/group. Similar results were obtained in an independent experiment measuring OVA‐specific IgM titers 4 days after i.v. immunization (data not shown). (B) Splenocytes of B6 mice 9 days after i.v. immunization with 1 μg of indicated mAb:OVA together with 25 μg αCD40 and 25 μg poly(I:C) were in vitro restimulated with I‐A^b^‐restricted OVA_262–276_. Cells were analyzed by flow cytometry using the gating strategy shown in Supporting Information Fig. 4. Percentages of IFN‐γ‐producing CD4**^+^** CD11a**^+^** T cells are shown as mean ± SEM of six independent experiments combined, using four to seven mice/group in each experiment. (C) Scheme for the experimental design of (D). (D) B6 mice were immunized according to the scheme in (C). High‐affinity NP‐specific IgG1 were analyzed by ELISA of 14 days after boost. Serum dilutions with OD > 0.1 are shown as mean ± SEM of a single experiment using five to six mice/group. (E, F) B6 mice infused with 4 × 10^5^ CD45.1**^+^** OT‐II CD4^+^ T cells were i.v. immunized with 1 μg of indicated mAb:OVA together with 25 μg αCD40 and 25 μg poly(I:C). (E) Tfh cells in spleen 10 days after immunization were analyzed by flow cytometry using the gating strategy shown in Supporting Information Fig. 5. Gates and numbers denote the frequency of CXCR5^+^ PD‐1^+^ Tfh cells. Gates are set on fluorescence minus one (FMO). Shown are dot plots of CXCR5 and PD‐1 expression on live, single, non‐AF, CD4^+^, CD45.1^+^ cells from one representative experiment from three independent experiments using three to five mice/group in each experiment. (F) Percentages of Tfh cells gated as shown in (E). Graph shows the mean ± SEM at day 10 (three experiments with three to five mice/group in each experiment) and day 28 after immunization (one experiment with three mice/group). (A, D) Data were analyzed by a Kruskal–Wallis test with Bonferroni's correction. *p*‐Value indicator # refers to *p* < 0.0167. (B, F) Data were analyzed by ANOVA with Bonferroni's correction; *p < 0.05 and **p < 0.005.

High‐affinity isotype‐switched Ab responses are classical signs of a CD4 Th‐cell‐dependent B‐cell activation. This was consistent with the nearly complete absence of detectable levels of anti‐OVA Abs after Ag targeting to DEC205^+^ DCs or CD169^+^ macrophages in MHC class II deficient mice (Fig. 3A). Furthermore, Ag targeting to DEC205^+^ DCs or CD169^+^ macrophages in WT mice led to the induction of OVA‐specific IFNγ‐producing CD4^+^ T cells (Fig. 3B, Supporting Information Fig. 4). The induction of T‐cell help could also be observed in a hapten‐carrier system. Mice were immunized with mAb:OVA and 28 days later boosted with 1 μg untargeted OVA‐NP_16_ (Fig. 3C). We observed a strong induction of high‐affinity αNP IgG1 after the boost when OVA was targeted to CD169^+^ macrophages during the primary immunization (Fig. 3D). These results suggest the efficient activation of Ag‐specific CD4 Th cells after Ag targeting to CD169^+^ macrophages.

To directly monitor OVA‐specific CD4^+^ T cells, we adoptively transferred OVA‐specific CD45.1^+^ OT‐II T cells to CD45.2^+^ mice and studied CD4^+^ T‐cell responses after immunization with αCD169:OVA or αDEC205:OVA. OVA targeting to CD169^+^ macrophages led to a higher percentage of OT‐II T cells with the CXCR5^+^ PD‐1^+^ Tfh phenotype at day 10 and day 28 postimmunization, compared to αDEC205 targeting (Fig. 3E and F, Supporting Information Fig. 5).

In conclusion, our results show that CD169^+^ macrophages mediated Ab responses are T cell dependent and that CD169^+^ macrophages targeting efficiently stimulates the activation of Ag‐specific Tfh cells.

### CD169^+^ macrophages retain intact Ag and upregulate adhesion molecules for B‐cell activation

**Figure 4:**
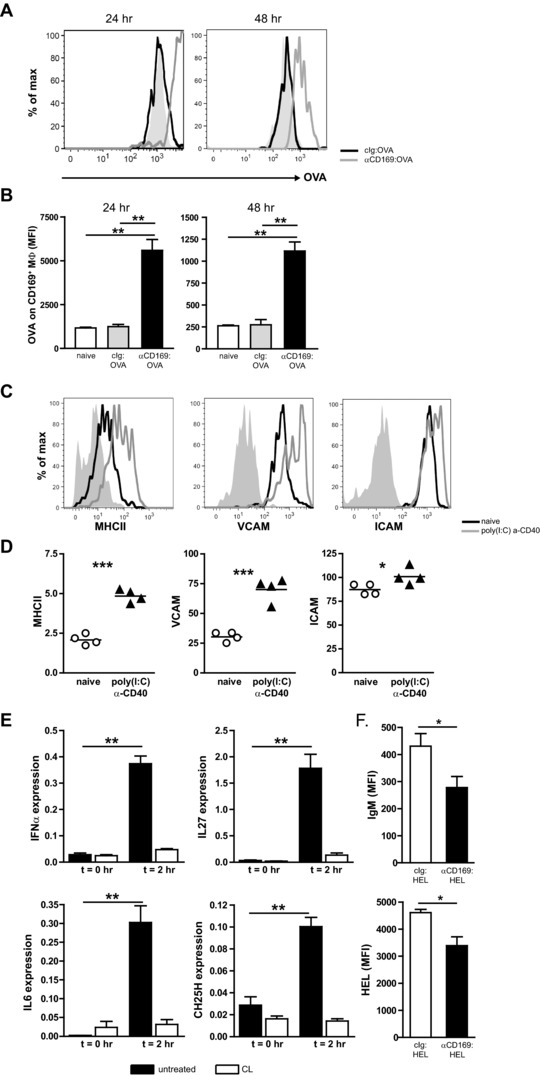
CD169^+^ macrophages retain intact Ag and upregulate adhesion molecules for B‐cell activation. (A, B) B6 mice were i.v. immunized with 20 μg of indicated mAb:OVA together with 25 μg αCD40 and 25 μg poly(I:C). Retention of OVA on splenic CD169^+^ macrophages was analyzed after 24 and 48 h by flow cytometry. (A) CD169^+^ macrophages were gated using the gating strategy shown in Supporting Information Fig. 6. Gates were set on FMO. Shaded gray histograms represent nonimmunized mice, black lines are cIg:OVA immunized mice, and gray lines are αCD169:OVA immunized mice. Representative histograms of anti‐OVA staining on live, AF, CD169^+^ cells from three (24 h) and two (48 h) experiments are shown. (B) Graph shows the geometric mean of the fluorescence intensity (mean ± SEM) of αOVA staining on CD169^+^ macrophages gated as described in (A). (C, D) B6 mice were immunized with 25 μg αCD40 and poly(I:C). Expression of indicated markers on live/dead marker^−^, AF^+^, CD169**^+^** cells was analyzed after 24 h. (C) Representative histograms showing expression levels of indicated markers on naïve (black line histogram) and adjuvant‐activated (gray line histogram) CD169**^+^** AF cells. Background staining (FMO) is depicted in shaded gray histogram. (D) Depicted is the ratio of the geometric mean of fluorescence intensity of the indicated markers, divided by the background staining from individual mice of one experiment out of two to four independent experiments using three to four mice/group in each experiment. (E) Untreated (black bars) or macrophage‐depleted mice (CL, white bars) were immunized with 25 μg αCD40 and poly(I:C). *Ifna, Il27, Il6*, and *Ch25h* mRNA levels in spleens at *t* = 0 h and 2 h were detected by qPCR. *Hprt* was used for normalization. Mean ± SEM from a representative experiment of two independent experiments using three mice/group per experiment is shown. (F) B6 mice were infused with CFSE‐labeled MD4 Tg cells and immunized with 1 μg mAb:HEL conjugate together with 25 μg αCD40 and poly(I:C). MD4 Tg B cells (live/dead marker^−^/ B220^+^/ CFSE^+^) were analyzed for cell surface IgM expression (upper) and HEL binding (bottom) 24 h after immunization using the gating strategy shown in Supporting Information Fig. 8. Data represent geometric mean of the fluorescence intensity ± SEM of the relevant marker of a single experiment with three to five mice/group. B220^+^, CFSE^+^ B cells were checked for the presence of IgDα^+^ in a separate staining to confirm that these cells are MD4 Tg B cells. (B, E) Data were analyzed by ANOVA with Bonferroni's correction. (D, F) Data were analyzed by a two‐tailed Student *t*‐test; *p < 0.05, **p < 0.005, and ***p < 0.0005.

Classically, macrophages are known for their high phagocytic function and degradative potential 35. In contrast, SCS CD169^+^ macrophages in LNs are characterized by a low proteolytic capacity, which allows the presentation of intact Ag to B cells. To investigate the presence of intact OVA bound to splenic CD169^+^ macrophages, we first identified CD169^+^ macrophages by flow cytometry as a small population of large, autofluorescent (AF), granular cells with high CD169, low CD11c, intermediate MHC class II, and high VCAM‐1 and ICAM‐1 expression (Supporting Information Fig. 6, Fig. 4C). These cells could be distinguished from a heterogeneous non‐AF population with lower CD169 expression that included IL‐7Rα^+^, CCR6^+^, and CD11c^high^ cells (Supporting Information Fig. 6B) that may have acquired CD169^+^ macrophages membrane blebs during the digestion, as recently described in LNs 36. Both CD169^+^ populations could not be detected in animals that lack CD169^+^ macrophages such as Ltα‐deficient mice and mice treated with CLs (Supporting Information Fig. 6C). However, only the AF^+^ CD169^+^ and not the AF^−^CD169^+^ population had bound Ag 30 min after targeting with CD169:OVA (Supporting Information Fig. 7). Interestingly, AF^+^ CD169^+^ macrophages still contained significant levels of OVA 24 and 48 h after αCD169:OVA injection (Fig. 4A and B). In addition, phenotypic analysis of AF^+^CD169^+^ macrophages showed a further increase in expression of both ICAM‐1 and VCAM‐1 after adjuvant injection. These molecules are known to facilitate the interaction with LFA‐1 and VLA‐4 on B cells and the capture of intact Ag by B cells 37, 38 (Fig. 4C and D). The upregulation of these molecules was dependent on the presence of adjuvants and was not seen when Ag was targeted to CD169^+^ macrophages without α‐CD40 and poly(I:C) (data not shown). Together these data demonstrate that CD169^+^ macrophages are able to present intact Ag for prolonged time and upon activation by adjuvants upregulate adhesion molecules for optimal interaction with B cells.

Next we studied whether macrophages in the marginal zone were required for the production of B‐cell stimulatory cytokines 39, 40, 41. Early production of IFN‐α,  IL‐27, and IL‐6 was detected in the spleen upon adjuvant injection (Fig. 4E, black bars) and these responses were abrogated when macrophages were specifically depleted from the marginal zone using CL treatment (Fig. 4E, white bars). Similarly, the enzyme CH25H, involved in the synthesis of 7α,25‐dihydroxycholesterol and important for migration of activated B cells, was induced after adjuvant injection and severely reduced after CL treatment (Fig. 4E). These results clearly suggest that the production of B‐cell stimulating factors is at least dependent on the presence of macrophages in the marginal zone.

To investigate cognate B‐cell activation after CD169^+^ macrophages targeting, we adoptively transferred hen egg lysozyme (HEL) specific MD4 Tg B cells into B6 mice. Preliminary experiments showed that immunization with αCD169:HEL resulted in reduced cell surface expression of IgM and reduced HEL binding to MD4 Tg B cells shortly after Ag targeting (Fig. 4F, Supporting Information Fig. 8), suggesting rapid activation induced endocytosis of the B‐cell receptor.

In conclusion, Ag targeting to CD169^+^ macrophages in the presence of adjuvant led to retention of intact Ag on these cells, to upregulation of costimulatory molecules that facilitate B‐cell activation, the production of B‐cell stimulating cytokines, and the activation of cognate B cells.

### Alternative targeting to CD169^+^ macrophages using the CR domain of the MR promotes Ab responses

**Figure 5:**
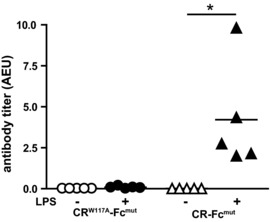
Ag targeting to CD169^+^ macrophages using the CR domain of MR promotes Ab responses. Mice were immunized with 4 pmol of cross‐linked CR‐Fc^mut^ or CR^W117A^‐Fc^mu^ with or without LPS. Human Fc‐specific Abs were analyzed 14 days after immunization by ELISA. Anti‐Fc Ab titers from individual mice are expressed as arbitrary ELISA units (AEU) of a representative experiment from two independent experiments with five to six mice/group per experiment. Data were analyzed by ANOVA with Bonferroni's correction; *p < 0.05.

The cysteine‐rich domain (CR) of the mannose receptor is known to bind to CD169 expressed by macrophages 42, 43, 44. We exploited the binding of the CR domain as a second strategy to target to CD169^+^ macrophages. We injected mice with recombinant CR‐Fc protein consisting of the CR domain of MR fused to a mutated version of human IgG1 Fc incapable of activating complement or binding to Fc receptors (CR‐Fc^mut^) in the presence and absence of LPS, and followed the α‐human Fc Ab response. CR‐Fc protein containing a single amino acid substitution that is unable to bind to CR ligands (CR^W117A^‐Fc^mut^) was used as a negative control 42. Robust Ab responses were observed when Ag was targeted to CD169^+^ macrophages via CR‐Fc^mut^ (Fig. 5), indicating that complement or Fc receptors are not involved in these processes. Similarly to the OVA‐specific Ab response, α‐human Fc Ab responses were not present in the absence of adjuvant. These data further underline our findings that targeting to CD169^+^ macrophages leads to strong humoral immunity that depends on cellular activation.

## Discussion

In this study we show that Ag targeting to CD169^+^ macrophages in the marginal zone of the spleen leads to isotype‐switched and high‐affinity Ab production via the promotion of GC activity. This GC response was CD4 T cell dependent and correlated with efficient induction of Tfh. We observed retention of intact Ag on CD169^+^ macrophages, upregulation of costimulatory molecules after activation, and presentation of Ag to cognate B cells. Our studies suggest that splenic CD169^+^ macrophages are able to induce potent humoral immunity via the promotion of GC B‐cell responses and identify these cells as interesting targets for vaccination strategies.

Several factors support the efficient induction of B‐cell immunity after Ag targeting to CD169^+^ macrophages. Our data demonstrate that splenic CD169^+^ macrophages retained intact Ag for at least 2 days (Fig. 4A and B), which is in line with a previous study that showed low proteolytic capacity of LN SCS CD169^+^ macrophages 32. The presence of intact Ag is requisite for B‐cell priming and our data indicate that Ag targeted to CD169^+^ macrophages is presented to cognate B cells (Fig. 4F). Furthermore, these findings could provide a possible mechanism for late‐arriving rare Ag‐specific B cells to encounter their cognate Ag, as been proposed for follicular DCs 45. Further studies employing intravital microscopy of the spleen will be necessary to demonstrate direct interaction of B cells with CD169^+^ macrophages in the marginal zone.

Importantly, splenic CD169^+^ macrophages are optimally equipped to activate B cells. They express high levels of ICAM‐1 and VCAM‐1, which are further upregulated after activation (Fig. 4C and D). These adhesion molecules enhance the formation of the immunological synapse between cognate B cells and Ag‐bearing cells and might support efficient interaction of CD169^+^ macrophages and naïve B cells 37, 38. Furthermore, we show that the production of B‐cell stimulating cytokines such as type I IFN, IL‐27, and IL‐6 was mediated by macrophages in the marginal zone (Fig. 4E) 39, 40.

Macrophages also mediated the induction of the enzyme CH25H (Fig. 4E) necessary for the synthesis of 7α‐25‐OHC, which is recognized by EBI2 and stimulates B‐cell migration to the outer follicular zone 46, 47, 48, 49. Although recent data demonstrated that CH25H is mainly produced by stromal cells 50, our data indicate that after immunization macrophages are necessary for the production of this enzyme. We speculate that oxysterol recognition by EBI2 may enhance macrophage B‐cell interaction by promoting B‐cell migration toward the marginal zone.

Our results indicate that Ag targeting to CD169^+^ macrophages led to enhanced numbers of Tfh cells (Fig. 3E and F). Tfh cells are crucial in the induction and maintenance of GC reactions (reviewed in McHeyzer‐Williams 51). How these increased Tfh responses are raised by Ag targeting to CD169^+^ macrophages is not clear. One possibility could be that Ag targeted to CD169^+^ macrophages is transferred to DCs, which in turn could be responsible for initial Tfh induction. Direct Ag targeting to DCs has been shown to efficiently activate Tfh and B‐cell responses 9, 12, 13, 52. We previously showed the existence of Ag transfer between CD169^+^ macrophages and DCs 33, thus it would be relevant to further study the role of DCs in CD169^+^ macrophage‐induced CD4^+^ T‐cell activation and humoral immunity.

A second possibility is that CD169^+^ macrophages directly stimulate Tfh differentiation. CD169^+^ macrophages express MHC II molecules and upon stimulation with adjuvant upregulate costimulatory molecules, which may enable Tfh cell activation. Ag persistence on DCs has been proposed to promote Tfh differentiation 52. Interestingly, we observed that CD169^+^ macrophages retained Ag for prolonged periods of time. However, since CD169^+^ macrophages are notoriously hard to isolate 36, we have not been able to directly test ex vivo presentation of Ag by CD169^+^ macrophages to CD4 T cells.

A third possibility is that the increased numbers of Tfh after Ag targeting to CD169^+^ macrophages could be the consequence of the very efficient Ag capture and stimulation of GC B cells. A recent study showed that the Tfh phenotype was maintained by sustained Ag presentation by GC B cells and is of transient nature 53. Our results clearly highlight the connection between Ag availability, GC B‐cell and Tfh response.

Overall, our study provides evidence that splenic CD169^+^ macrophages are capable of inducing high‐affinity, isotype‐switched Ab responses after Ag targeting. This capacity of splenic CD169^+^ macrophages to stimulate humoral immunity correlates with prolonged presentation of intact Ag, with expression of costimulatory molecules and induction of Tfh. Since also strong CD8**^+^** T‐cell and iNKT responses are obtained after Ag targeting to splenic CD169^+^ macrophages  28, 33, Ag targeting to CD169^+^ macrophages appears to potently activate both cellular and humoral immune responses and provides a promising new vaccination strategy.

## Materials and methods

### Preparation of mAb‐OVA and mAb‐HEL conjugates

Purified rat IgG2a Ab αCD169 (MOMA‐1), α‐DEC205 (NLDC145), and isotype control (R7D4) were activated with the cross‐linker SMCC (Pierce). LPS‐free OVA (Seikagaku) and HEL (Sigma‐Aldrich) proteins were functionalized with SATA (Pierce). After removal of excess reagent and deacetylation of SATA, Abs were conjugated either with five equivalents (molar ratios) of OVA or HEL protein. Unconjugated OVA or HEL protein was removed by gel filtration using Superdex 200 column (Amersham).

### Mice

C57Bl6/J, MHC‐II‐deficient, OT‐II, CD45.1 congenic mice were obtained from Charles River or the Jackson Laboratory and bred at the animal facility of the VU University Medical Center. Balb/c mice were bred at the animal facility of the Oxford University (Oxford, UK). All mice used in this study were matched for age and sex and kept under specific pathogen‐free conditions. Experiments were approved by the Animal Ethics Committee of our institutes.

### Immunizations

Mice were immunized i.v. with 1 μg mAb:OVA in the presence or absence of 25 μg poly(I:C) and 25 μg αCD40 Ab 1C10 54. When described mice were boosted with 1 μg OVA NP_16_ (Biosearch Technologies) i.v. 28 days after primary immunization.

To deplete CD169^+^ macrophages, mice were injected i.v. with 200 μl clodronate containing liposomes and immunized as described above 7–8 days after liposome injection. In this time frame DCs and red pulp macrophages have repopulated the spleen but macrophages in the marginal zone are still depleted (34 and Supporting Information Fig. 2).

For adoptive transfer of 4 × 10^5^ OT‐II T cells, CD4^+^ T cells were purified from spleen and LNs of CD45.1^+^ OT‐II Tg mice by negative depletion using a magnetic‐bead CD4^+^ T‐cell isolation kit (Dynal Biotec ASA) according to the manufacturers’ protocol.

For adoptive transfer of MD4 Tg B cells, spleen, and LN cells of IghelMD4 Tg mice (kindly provided by Dr. E. Eldering, Academic Medical Center, The Netherlands) were labeled with 5 mM CFSE for 5 min at 37°C. Mice were injected i.v. with 5 × 10^6^ cells and immunized with 1 μg mAb:HEL together with 25 μg αCD40 and 25 μg poly(I:C).

For flow cytometric analyses of OVA uptake after immunization, mice were injected i.v. with 20 μg mAb:OVA together with 25 μg αCD40 and 25 μg poly(I:C). For flow cytometric analyses of phenotypical changes of CD169^+^ macrophages  mice were injected i.v. with 25 μg αCD40 and 25 μg poly(I:C).

In some experiments, humoral responses were analyzed at day 14 after i.v. immunization with 4 pmol of cross‐linked CR‐Fc^mut^ or CR^W117A^‐Fc^mut^ in the presence and absence of 5 μg LPS. Cross‐linking was achieved with mouse F(ab)′2 α‐human Fc 42. To generate hyperimmune serum Balb/c mice were immunized s.c. three times with 20 μg hIgG1 in the presence of CFA and incomplete Freund's adjuvant.

### Anti‐OVA, anti‐NP Ab, and anti‐human Fc Ab ELISA

High‐binding 96‐well plates (Nunc Maxisorp) were coated with 5 μg/mL OVA (Sigma‐Aldrich), 5 μg/mL NP_4_‐BSA (Biosearch Technologies), or 10 μg/mL EGF5‐6‐Fc in PBS and blocked with 1–3% BSA in PBS. Serial dilutions of serum in 1% BSA/PBS were incubated for 1–2 h at RT. Detection was achieved using polyclonal rabbit α mouse Ig‐HRP (Dako), biotin‐labeled α mouse IgM, IgG1, IgG2b, or IgG3 (all Dako) followed by streptavidin‐conjugated HRP (Jackson Immune Research), or alkaline phosphatase conjugated α‐mouse IgG. Ab titers were determined as the dilution that resulted in an OD450 of more than 0.1. For the α‐human Fc ELISA, serum from mice hyperimmunized with hIgG1 was used as a positive control and internal standard.

For the anti‐OVA Ab avidity ELISA 55 ammonium thiocyanate (NH_4_SCN) at concentrations ranging from 0 till 4 M was added to the wells for 15 min directly after sample incubation and the assay was finished using polyclonal rabbit α mouse Ig‐HRP. The avidity index was calculated as the concentration of NH_4_SCN, which resulted in an OD_50_ of the wells where no NH_4_SCN was added.

### Ex vivo CD4^+^ T‐cell restimulation assay

Splenocytes from mice 9 days after i.v. immunization with 1 μg mAb:OVA together with 25 μg poly(I:C) and 25 μg αCD40 were restimulated in vitro for 18 h with MHC class II restricted OVA_262–276_ peptide (100 μg/mL) followed by 5 h incubation with GolgiPlug (BD Biosciences). Cells were analyzed for intracellular cytokine expression by flow cytometry.

### Flow cytometry

For CD169^+^ macrophages isolation, spleens digested with 1 Wu/mL liberase TL (Roche Diagnostics), 4 mg/mL lidocaine hydrochloride monohydrate (Sigma), and 50 μg/mL DNAse (Roche Diagnostics) in PBS at 37°C. Staining was performed with mAbs listed in Supporting Information after blocking Fc receptor with clone 2.4G2. All Abs for extracellular stainings were diluted at the appropriate concentration in PBS containing 0.5% BSA. Abs were incubated for 30 min at 4°C. GC B cells were detected with the use of 5 μg/mL OVA‐488 (Invitrogen) incubated together with the primary Abs. For intracellular staining (Figs. 3B, 4A, and B) cells were fixed in PBS containing 2% paraformaldehyde and stained subsequently in PBS supplemented with 0.5% BSA and 0.5% saponine. Sytox blue nucleic acid stain (Invitrogen) or LIVE/DEAD® Fixable Near‐IR Dead Cell Stain Kit (Invitrogen) were used according to the manufacturers’ protocol. Cells were analyzed using a Cyan ADP flow cytometer (Beckman Coulter) and the Flowjo software package (Tree Star).

### Real‐time PCR

Total RNA was isolated using TRIzol reagent (Invitrogen Life) and precipitated with isopropanol. cDNA was synthesized using RevertAid First Strand cDNA Synthesis Kit (Fermentas Life Sciences) according to manufacturers’ protocol. Real‐time PCR was performed using SYBR Green Mastermix on an ABI Prism 7900HT Sequence Detection System (PE Applied Biosystems). A standard curve was generated using pooled LN tissue to correct for primer efficiency. mRNA quantities were normalized to HPRT. Primers are listed in Supporting Information.

### Statistical analysis

Statistical significance was tested using GraphPad Prism 4 or SPSS by performing a two‐tailed Student *t*‐test, ANOVA with Bonferroni's correction, or a Kruskal–Wallis test with Bonferroni's correction.

## Conflict of interest

The authors declare no commercial or financial conflict of interest.

**Abbreviations**
AEU: arbitrary ELISA units
AF: autofluorescent
CL: clodronate liposome
CR: cysteine‐rich domain
FMO: fluorescence minus one
HEL: hen egg lysozyme
NH_4_SCN: ammonium thiocyanate
SCS: subcapsular sinus
Tfh: follicular Th cell

## Supporting information

As a service to our authors and readers, this journal provides supporting information supplied by the authors. Such materials are peer reviewed and may be re‐organized for online delivery, but are not copy‐edited or typeset. Technical support issues arising from supporting information (other than missing files) should be addressed to the authors.

Figure 1
Figure 2
Figure 3
Figure 4
Figure 5
Figure 6
Figure 7
Figure 8

Peer review correspondence
