# Supplementary material for: Antigen targeting reveals splenic CD169+ macrophages as promoters of germinal center B‐cell responses
Source: Eur J Immunol. 2015 Jan 14;45(3):747–57. doi: 10.1002/eji.201444983 (PMC4843951; doi:10.1002/eji.201444983)
Supplement: Supplementary file 1 — Figure 1 Figure 2 Figure 3 Figure 4 Figure 5 Figure 6 Figure 7 Figure 8 [file EJI-45-747-s001.pdf]

European Journal of Immunology

Supporting Information
for
DOI 10.1002/eji.201444983

Henrike Veninga, Ellen G. F. Borg, Kyle Vreeman, Philip R. Taylor, Hakan Kalay, Yvette van Kooyk, Georg Kraal, Luisa Martinez-Pomares and Joke M.M. den Haan

Antigen targeting reveals splenic CD169^{+} macrophages as promoters of germinal center B-cell responses

© 2014 WILEY-VCH Verlag GmbH & Co. KGaA, Weinheim

SUPLLEMENTARY INFORMATION

Antibodies

All antibodies were obtained from eBioscience, unless otherwise indicated.

GC B cells: OVA-488 (Invitrogen), GL7 (GL7), B220 (6B2), CD38 (90, AbD Serotec), SA-PerCP (BD Pharmingen), sytox blue nucleic acid stain (Invitrogen)

IFNγ producing CD4 T cells: CD11a (M17/4), CD4 (L3T4), IFNγ (XMG1.2)

Tfh cells: CD45.1 (A20), CD4 (GK1.5, own production), CXCR5 (614641 RnD Systems), PD-1 (J43), BCL6 (K112-91, BD Pharmingen), SA-PeCy7, SA-Alexa Fluor 647 (Invitrogen), or SA-APC eFluor 780, sytox blue nucleic acid stain

$CD169^+$ Mφ: CD169 (Ser4 or Moma-1, own production), LIVE/DEAD® Fixable Near-IR Dead Cell Stain Kit (Invitrogen), CD11c (N418), VCAM-1 (429), ICAM-1 (YN1/1.7.4), MHCII (M5/115, own production), CCR6 (own production), IL7Rα (A7R34, own production), donkey anti rat PE (Jackson ImmunoResearch), polyclonal rabbit anti-OVA (ICN pharmaceuticals), donkey anti-rabbit IgG Alexa Fluor 647 (Invitrogen), or donkey anti rabbit IgG R-PE (Jackson ImmunoResearch), goat anti rat IgG 555 (Invitrogen)

MD4 tg B cells: B220 (6B2), IgDα (AMS9.1, BD Pharmingen), hen egg lysozyme (HEL, Sigma), α-chicken lysozyme (rabbit polyclonal, MyBioSource.com), goat anti-rabbit IgG Alexa Fluor 647 (Invitrogen), IgM (II/41, BD Pharmingen), SA-PeCy7

Immunofluorescence stainings: CD169 (Moma-1), CD169:OVA (Moma-1), anti-rat IgG-Alexa 488, or rabbit anti-OVA, anti-rabbit IgG-Alexa 488, CD11c (N418)

DEC205 on DC subsets: CD11c (N418), CD8α ((53-6.7), cIg (R7D4), DEC-205, cIg:OVA and DEC-205:OVA, anti-rat IgG-Alexa 488 or polyclonal rabbit anti-OVA, and anti-rabbit IgG-Alexa 488

Primer sequences

IFNα F: GAG AGA AGA AAC ACA GCC CC

IFNα R: TGC TCT GAC CAC CTC CC

IL6 F: GAG TTG TGC AAT GGC AAT TCT G

IL6 R: TGG TAG CAT CCA TCA TTT CTT TGT

IL27 p28 F: GCAGGGAATTCACAGTCAG

IL27 p28 R: AGTCAGGGAAACATTGGGA

SUPLLEMENTARY MATERIALS AND METHODS

Immunofluorescence

Spleen fragments were embedded in O.C.T. (Tissue-Tek, Sakura) and 5 µm cryosections were fixed in dehydrated acetone for 5 min. and air-dried. Sections were blocked with 10% mouse serum in PBS for 15 min. prior to staining. Sections were stained with antibodies listed above and analyzed using a Leica DM6000 microscope.

OVA ELISA

High binding 96 well plates (Nunc Maxisorp) were coated with serial dilutions (1:3) starting with 1 µg/ml mAb:OVA in PBS for 1 hr at 37 °C and blocked with 1% BSA in PBS. Detection was achieved using 1:1000 diluted polyclonal rabbit anti-OVA in 1% BSA in PBS followed by anti-rabbit Ig conjugated HRP (Jackson Immune Research), both incubated for 1 hr. In between all steps plates were was intensively with PBS containing Tween.

Ex vivo CD8$^+$ T cell restimulation assay

Splenocytes from mice 9 days after i.v. immunization with 1 µg mAb:OVA together with 25 µg poly(I:C) and 25 µg αCD40 with or without treatment with clodronate liposomes 7-8 days prior to immunization were restimulated *in vitro* for 5 hrs with MHC class I restricted $OVA_{257-264}$ peptide (100 ng/ml) in the presence of GolgiPlug (BD Biosciences). Cells were analyzed for intracellular cytokine expression by flow cytometry.

SUPPLEMENTARY FIGURE LEGENDS

Fig. S1: mAb:OVA complexes retain antigen specificity and contain similar amounts of OVA. (A) Spleen sections were incubated with 10 ug/ml αCD169 or αCD169-OVA for 30 minutes and detected with anti-rat IgG-Alexa 488 Ab (green) or rabbit anti-OVA followed by anti-rabbit IgG-Alexa 488 Ab (green). $CD11c^+$ DCs were detected by anti-CD11c staining (red). Shown are representative pictures of a single experiment using 1 mouse. Background staining with cIg:OVA was hardly present. (B) Splenocytes were incubated with 10 ug/ml cIg, αDEC205, cIg:OVA, or αDEC205:OVA for 30 minutes. Bound cIg, αDEC205, cIg:OVA and αDEC205:OVA were detected by incubation with anti-rat IgG-Alexa 488 or by polyclonal rabbit anti-OVA followed by anti-rabbit IgG-Alexa 488. Shown are representative histograms gated on $CD11c^+CD8^+$ DCs from two independent experiments using one mouse. Grey histogram is cIg binding, black lines represent αDEC205 binding. (C) mAb:OVA complexes were coated on Maxisorb plates in serial dilution (start 1 ug/ml, 1:3 titrations) and evaluated for the presence of OVA by ELISA. Depicted is the mean ± SEM of the OD450 of a representative experiment from three independent experiments using three replicates/group.

Fig. S2: CL treatment specifically affects macrophages in the marginal zone. (A) Sections of spleens either non-treated (B6) or 7-8 days after clodronate liposome treatment (CL) were stained with anti-CD169 (green) and B220 (red). Shown are representative pictures of two independent experiments using one to three mice/group. (B) Flow cytometric analysis of DC subsets in spleen 7-8 days after clodronate liposome treatment (CL) or no treatment (B6). Shown are dot plots of CD11c and CD8 expression (left plots) on live, single, non-AF cells and CD4 and CD8

expression (right plots) on CD11c high expressing cells from two independent experiments using one to three mice/group. Gates and numbers denote the frequency of cells. Gates are set on FMO. (C) Percentage of IFNγ producing CD8+ (left graph) and $CD4^+$ (right graph) $CD11a^+$ T cells after *in vitro* restimulation with MHC I $OVA_{257-264}$ peptide or MHC II $OVA_{262-276}$ peptide respectively in spleen of B6 mice 9 days after i.v. immunization with 1 µg of indicated mAb:OVA together with 25 µg αCD40 and 25 µg poly(I:C). Mice were either untreated (white bars) or i.v. injected with clodronate liposomes (CL) (black bars) 7-8 days prior to immunization to deplete Mφ from the marginal zone. Graph shows mean ± SEM of two independent experiments combined using five to six mice/group. Gating strategy is shown in Fig. S4. Data were analyzed by ANOVA with Bonferroni's correction. P-value indicator ** refers to $P < 0.005$.

Fig. S3: Flow cytometry of GC B cells. (A) Gating strategy of GC B cells. Cells were first gated on the basis of forward and side scatter, live cells were identified as sytox $blue^-$. Single cells were gated by pulse width. Autofluorescent cells were gated out using an empty channel from the violet laser. B cells were defined as B220+ and GC B cells as B cells that had no expression of CD38 and stained for GL7. Gate were set on FMO. Shown are representative dot plots of six experiments using five to seven mice/group.

Fig. S4: Flow cytometry of IFNγ producing CD4+ T cells. (A) Gating strategy of IFNγ producing CD4+ T cells. Cells were first gated on the basis of forward and side scatter, single cells were gated by pulse width. Autofluorescent cells were gated out using an empty channel from the violet laser. CD4+ T cells were defined as CD4+ and IFNγ producing was evaluated against CD11a expression. Shown are representative dot plots of six experiments using four to seven mice/group.

Fig. S5: Flow cytometry of OT-II T cells. (A) Gating strategy of OT-II T cells. Cells were first gated on the basis of forward and side scatter, live cells were identified as FVD-. Single cells were gated by pulse width. Autofluorescent cells were gated out using an empty channel from the violet laser. CD4+ T cells were defined as CD4+, and OT-II cells were defined as CD45.1+. Shown are representative dot plots of four experiments using three to five mice/group.

Fig. S6: Flow cytometry of AF CD169$^+$ Mφ and non-AF CD169$^+$ cells.

(A) Gating strategy of AF CD169$^+$ cells and non-AF CD169$^+$ cells. Cells were first gated on the basis of forward and side scatter, live cells were identified as sytox blue$^-$. CD169 expression was evaluated against an empty channel from the violet laser to visualize autofluorescence. Shown are representative dot plots and histograms of five experiments using at least three mice/group. (B) Shown are representative dot plots and histograms of a single experiment using three mice/group. AF CD169$^+$ cells are large and granular and do not express CCR6 or IL7Rα and have low expression of CD11c. Non-AF CD169$^+$ cells are small and non-granular and contain cells positive for CCR6 or IL7Rα and exhibit a heterogeneous expression of CD11c and include CD11c^{high} cells. (C) AF and non-AF CD169$^+$ cells are absent in lymphotoxin α (Ltα) deficient mice and in mice treated with clodronate liposomes (CL). Shown are representative dot plots of a single experiment using three mice/group.

Fig. S7: OVA is targeted to CD169$^+$ Mφ and not present on non-AF CD169$^+$ cells. B6 mice were immunized with 1 μg of indicated mAb:OVA complexes together with 25 μg αCD40 and poly(I:C) and analyzed for OVA localization by flow cytometry 30 min after

injection. CD169$^+$ AF and non-AF cells were gated as described in Fig. S6. Shown is the geometric mean fluorescence intensity (mean ± SEM) of anti-OVA staining on AF CD169$^+$ and non-AF CD169$^+$ cells. Data are from a single experiment with three mice/group. Similar data was obtained in an independent experiment using 1 µg of indicated mAb:OVA complexes without adjuvants which was analyzed 5 min after immunization (data not shown).

Fig. S8: Analysis of mAb:HEL complexes and flow cytometry of MD4 B cells. (A) Spleen sections were incubated with 5 ug/ml αCD169 or αCD169-HEL for 30 minutes and detected with anti-rat IgG-Alexa 647 Ab (green) or rabbit anti-HEL followed by anti-rabbit IgG-Alexa 647 Ab (green). CD11c$^+$ DCs were detected by anti-CD11c staining (red). Shown are representative pictures of a single experiment using 1 mouse. Background staining with cIg:OVA was hardly present. (B) mAb:HEL conjugates were coated on Maxisorb plates in serial dilution (start 3 ug/ml, 1:3 titrations) and evaluated for the presence of HEL by ELISA. Depicted is the mean ± SEM of the OD450 of a representative experiment from two independent experiments using three replicates/group. (C,D) B6 mice were immunized with 1 µg or 20 µg of indicated mAb:HEL complexes and spleens were analyzed for HEL localization by flow cytometry 30 min after injection. Shown are histograms gated on CD169+ AF+ cells (C) and graphs with the geometric mean fluorescence intensity of anti-HEL staining (D). Grey histogram is cIg binding, black lines represent αCD169:HEL binding. CD169$^+$ AF cells were gated as described in Fig. S6. Data are from a single experiment with one mice/group. (E) B6 mice were infused with CFSE labeled MD4 transgenic cells and immunized with 1 µg mAb:HEL conjugate together with 25 µg αCD40 and poly(I:C). MD4 tg B cells were analyzed 24 hr after immunization. Shown is the gating strategy of MD4 B cells. Cells were first gated on the basis of forward and side scatter, live cells

were identified as sytox-blue-. Single cells were gated by pulse width. Autofluorescent cells were gated out using an empty channel from the violet laser. B cells were defined as B220+ and MD4 B cells were defined as CFSE+. Shown are representative dot plots of two independent experiments using three to five mice/group. (F) IgM expression and HEL binding were analyzed on MD4 tg B cells gated as described in (E). Shown are representative histograms of a single experiment using three to five mice/group. Shaded gray histograms represent non-immunized mice, grey lines are cIg:HEL immunized mice, and black lines are αCD169:HEL immunized mice.

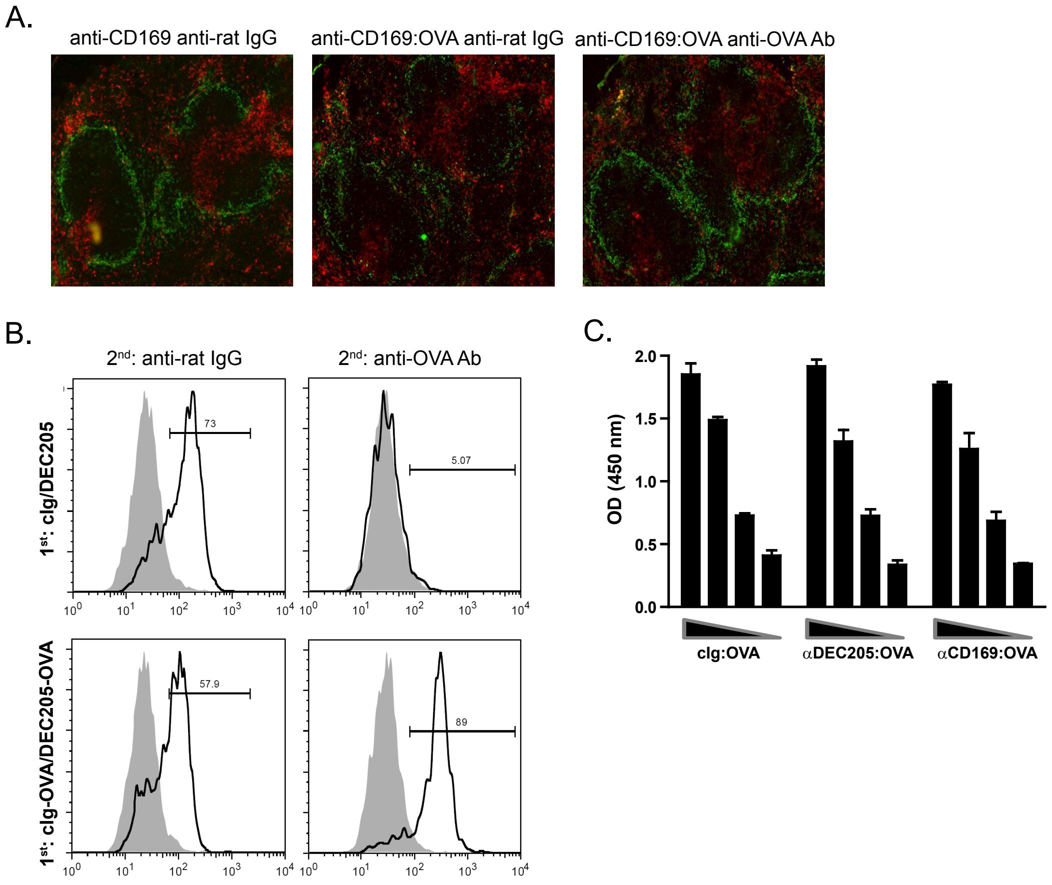

Veninga *et al*.
Supplementary figure 1

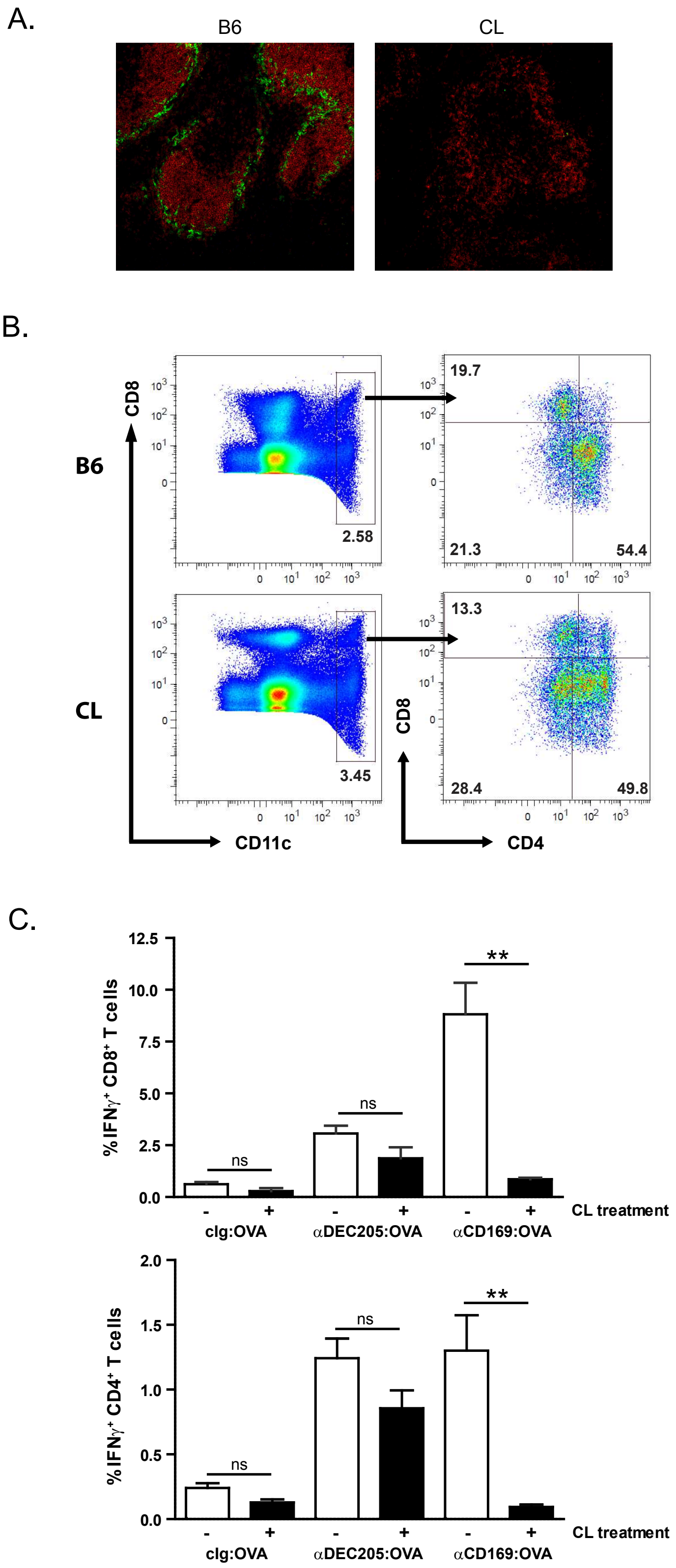

Veninga *et al.*
Supplementary figure 2

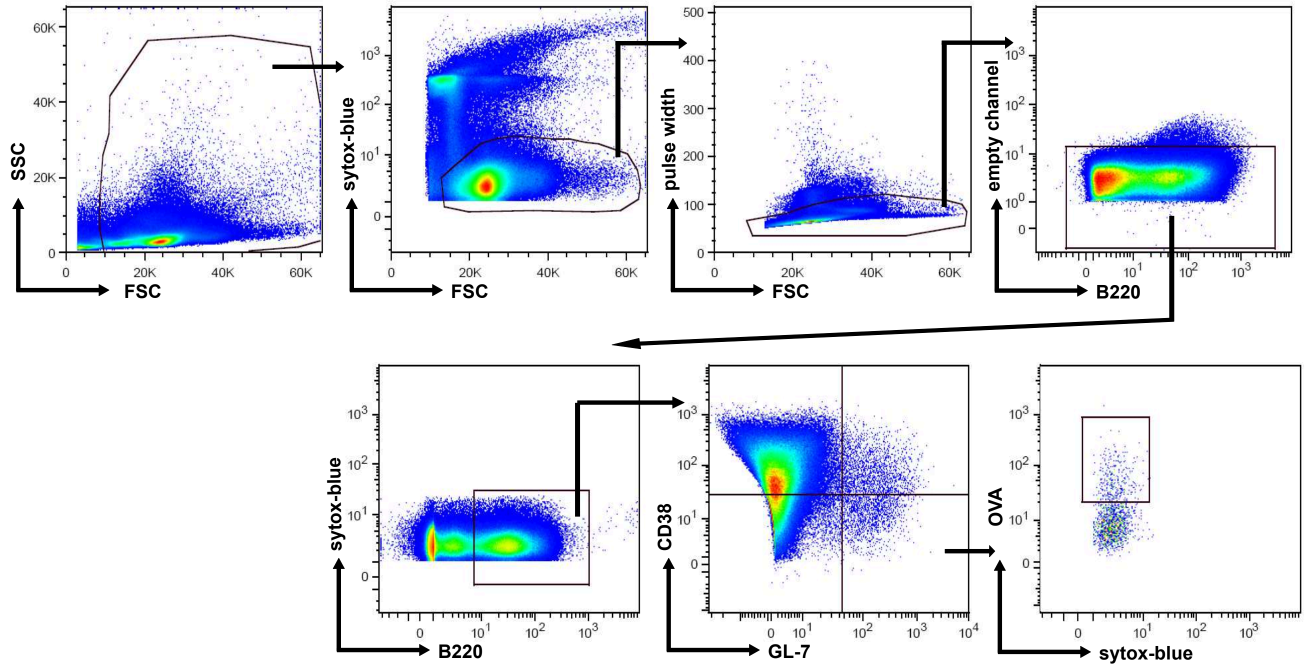

Veninga *et al*.
Supplementary figure 3

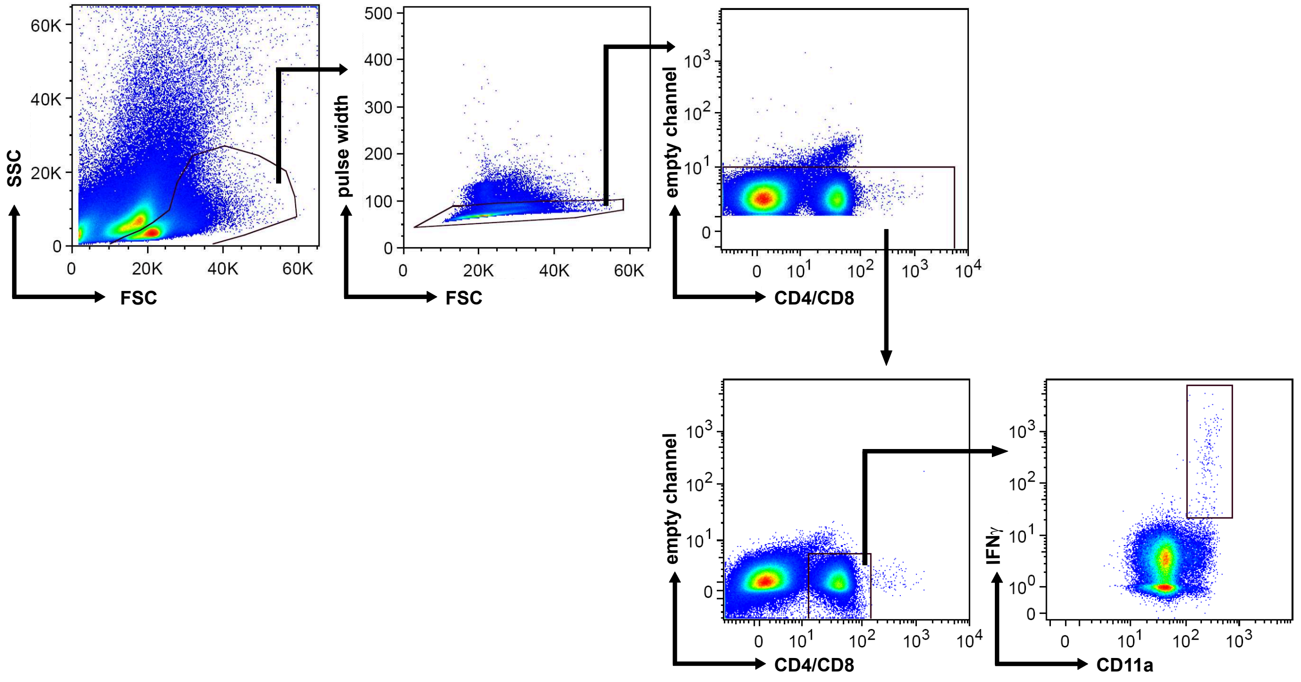

Veninga *et al.*
Supplementary figure 4

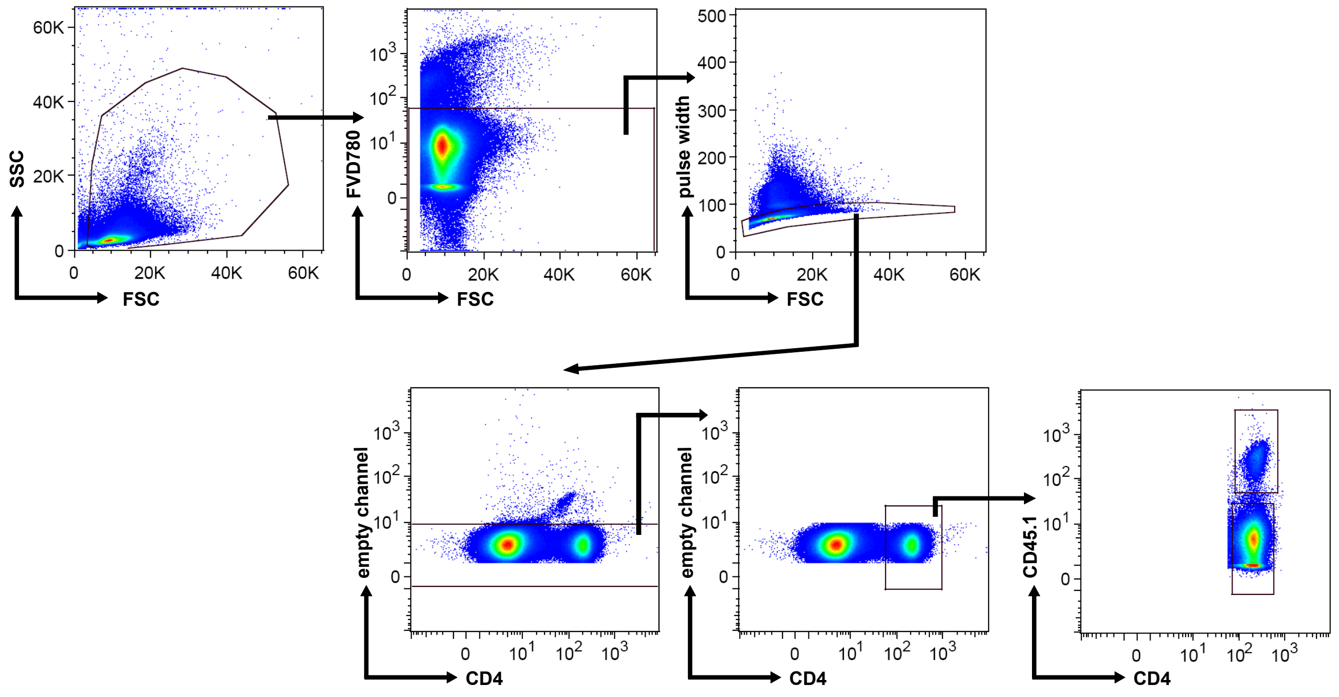

Veninga *et al.*
Supplementary figure 5

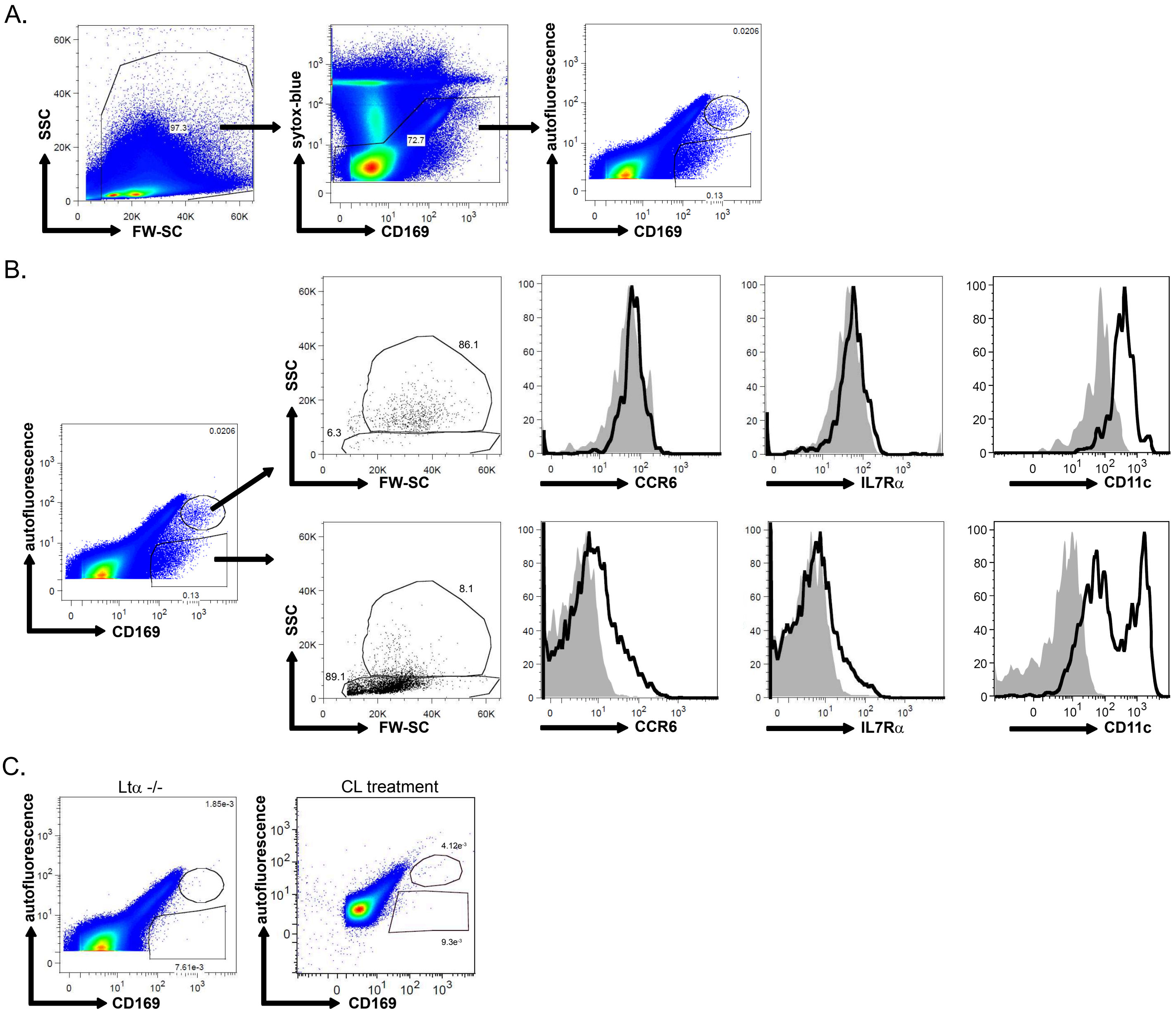

Veninga *et al.*
Supplementary figure 6

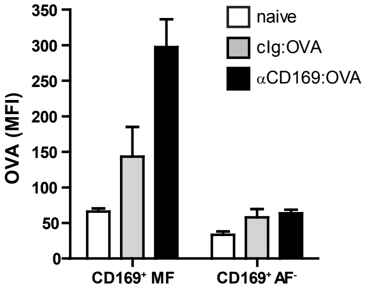

Veninga *et al*.
Supplementary figure 7

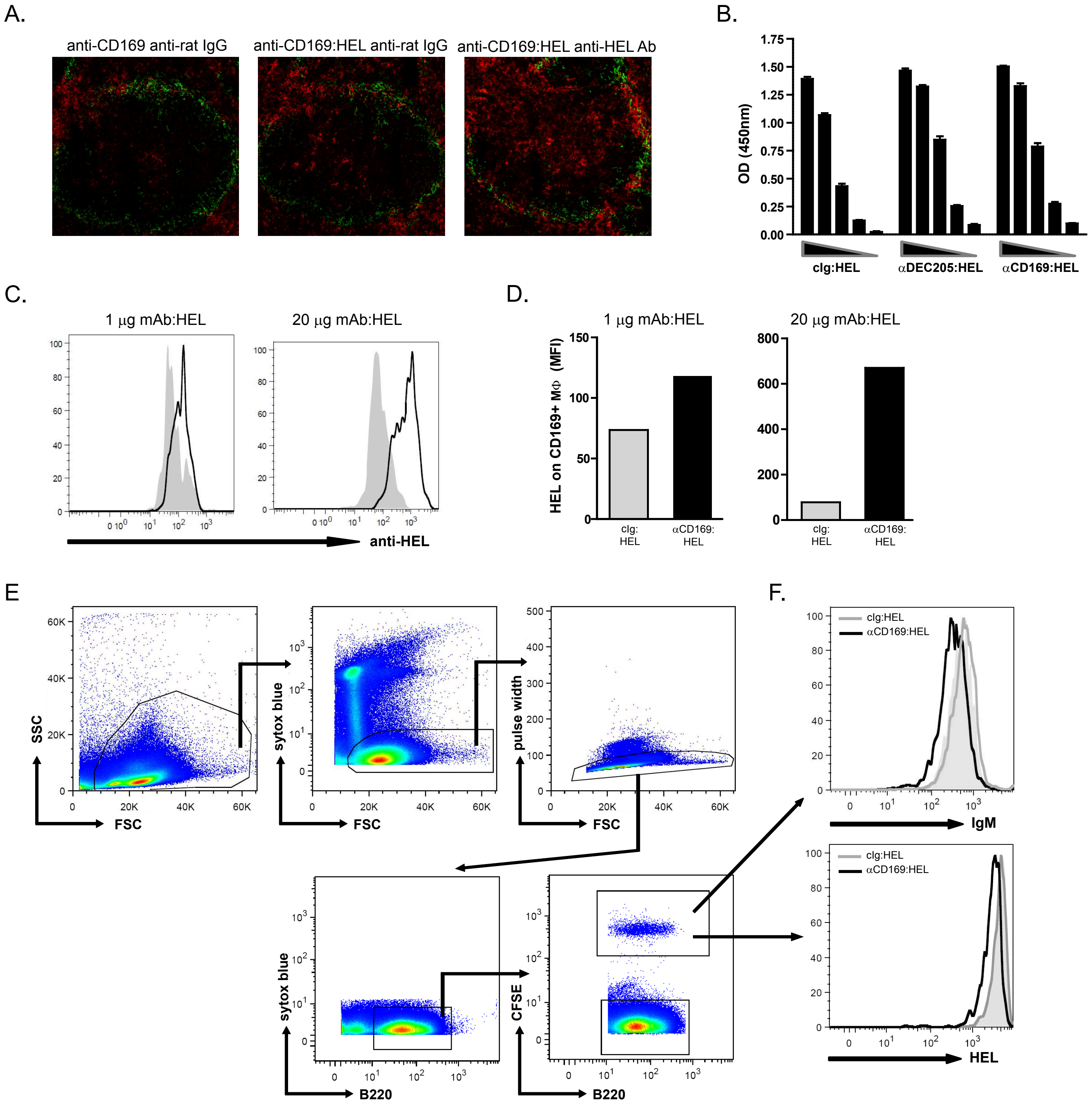

Veninga *et al.*
Supplementary figure 8
