# Supplementary material for: Antigen targeting reveals splenic CD169+ macrophages as promoters of germinal center B‐cell responses
Source: Eur J Immunol. 2015 Jan 14;45(3):747–57. doi: 10.1002/eji.201444983 (PMC4843951; doi:10.1002/eji.201444983)
Supplement: Supplementary file 2 — Peer review correspondence [file EJI-45-747-s002.pdf]

European Journal of Immunology

Peer review correspondence

Antigen targeting reveals splenic CD169^{+} macrophages as promoters of germinal center B-cell responses

Henrike Veninga, Ellen G.F. Borg, Kyle Vreeman, Philip R. Taylor, Hakan Kalay, Yvette van Kooyk, Georg Kraal, Luisa Martinez-Pomares and Joke M.M. den Haan

Corresponding author: Joke M.M. den Haan, Department of Molecular Cell Biology and Immunology, VU University Medical Center, Amsterdam, The Netherlands

---

Review Timeline:	Submission date:	1 July 2014
	Editorial decision:	4 August 2014
	Revision received:	21 October 2014
	Accepted:	3 December 2014

Handling Executive Committee member: Dr. Steffen Jung

Please note that the correspondence below does not include the standard editorial instructions regarding preparation and submission of revised manuscripts, only the scientific revisions requested and addressed.

First Editorial Decision – 4 August 2014

Dear Dr. den Haan,

Manuscript ID eji.201444983 entitled "Antigen targeting reveals splenic CD169+ macrophages as promoters of T cell dependent germinal center B cell responses" which you submitted to the European Journal of Immunology has been reviewed. The comments of the referees are included at the bottom of this letter.

A revised version of your manuscript that takes into account the comments of the referees will be reconsidered for publication.

You should also pay close attention to the editorial comments included below. In particular, please edit your figure legends to follow Journal standards as outlined in the editorial comments. Failure to do this will result in delays in the re-review process.

European Journal of Immunology

Please note that submitting a revision of your manuscript does not guarantee eventual acceptance, and that your revision will be re-reviewed by the referees before a decision is rendered.

If the revision of the paper is expected to take more than three months, please inform the editorial office. Revisions taking longer than six months may be assessed by new referees to ensure the relevance and timeliness of the data.

Once again, thank you for submitting your manuscript to European Journal of Immunology and we look forward to receiving your revision.

Yours sincerely,
Karen Chu

On behalf of Dr. Steffen Jung

Dr. Karen Chu
Editorial Office
European Journal of Immunology
e-mail: ejied@wiley.com
www.eji-journal.eu

****

Reviewer: 1

Comments to the Author
The manuscript by Veninga et al. tackles an interesting and actual subject that is to target the CD169+ macrophage population, present in secondary lymphoid organs, for vaccination purposes. Previous work from several labs, including authors of this work, showed the particular abilities of these macrophages to capture antigen travelling in the lymph, and present it intact to B cells or even transfer it to other cells such as B cells or DC. In the present manuscript, the authors compared the T-dependent B cell immune responses of mice immunized with antigens targeted to CD169+ macrophages or to DC, using antigen conjugated to antibodies against CD169 or to DC205 (DC marker). They showed that the immune responses are stronger and long-lasting when the antigen is targeted to CD169+ macrophages than when it is targeted to DC. They described that CD169+ macrophages, in addition to retain antigen, are important for other events like the production of cytokines and enzymes crucial to promote B cell responses. The manuscript is well written, the experiments are well performed, and the conclusions are supported by the

European Journal of Immunology

shown data, and of relevance for immunologist community. I have however some major concerns that the authors should address.

1. The authors claim in the title and along the manuscript that these macrophages are “promoters of T cell dependent germinal centre B cell responses”. They used anti-CD40 antibody as adjuvant in practically the whole experimentation (except for Fig 5), which will bias towards a T cell dependent response already. I wonder what would happen in absence of anti-CD40 as part of the adjuvant, the CD169+ macrophages would promote a T cell dependent or T cell independent response at the spleen?? What about the comparison with the DC-targeted response in that case?? The authors should clarify this issue in the title and text, highlighting that the study is just a comparison between macrophage and DC abilities to induce a T-dependent humoral immune response when the antigen is targeted to one or the other cell type, or perform the needed experiments to answer the questions mentioned above.

2. How does the antigen retention ability of CD169+ macrophages compare to that of DC at the times analysed? …taking into account that a GC reaction takes 7 days more or less to form, and the role of antigen transfer from CD169+ macrophages to FDC (through B cells) for affinity maturation (Phan TG et al., Nat Immunol 2009), what about antigen retention at longer times than 48h at the surface of these cells?

3. Is the upregulation of adhesion molecules and class II at macrophage surface similar when only adjuvant is administered to mice, in absence of OVA-conjugated anti-CD169 antibody??

4. The elimination of macrophages with clodronate liposomes resulted in no production of the B cell stimulating factors IL-6, IL-27 and IFNa at the time analysed (2h) …….do the authors detect any sign of cytokine expression at later time points??.... is it just a matter of delayed kinetic or the spleen is not showing any sign of immune response at all (cytokine secretion, B cell activation, Ab production....)??

Reviewer: 2

Comments to the Author

The manuscript by Veninga et al. dissects the role of CD169 macrophages in promoting CD4 T cell and B cell responses. The paper is methodical and well written. I only have minor comments and request the repetitions of key experiments. If the repetition of these experiments is not feasible, I would like the authors to highlight in the discussion that their conclusions are based on “one-off” experiments (and are indicative rather than conclusive).

European Journal of Immunology

Figure 1A shows OVA-specific responses in mice injected with 1 ug Ig, a-CD169 or a-DEC205 in the presence of 25 ug aCD40 + 25 ug polyIC. On day 9, naïve and cIg immunized mice have an 1/1000 titer. By contrast, aDEC or aCD169 targeted mice have 1/5000 or 1/6000 titers. Thus targeted mice have 5-6 times higher responses. In Figure 1C there appears to be approximately 10-fold difference between targeted and non-targeted. However, in Figure 1D, in mice immunized under the same conditions, there is only a 2-fold difference between targeted delivery and non-targeted delivery: the cIg-OVA: OD 0.5 and aDEC or a-CD169 give an OD 1.00. It appears that targeting is barely advantageous in this experiment only providing a 2-fold enhancement. If that is the case maybe it was not the best experiment to deplete macrophages in, since targeting did not seem to work well.

Figure 4F – what are the units of the y-axis; Fig. 4A and C lack legends.

The authors have conjugated HEL to mAb. Does this conjugate retain the ability to recognize CD169 and is the conjugated HEL still recognized by MD4 transgenic? I would like to see data that the conjugate bind its target and that this binding was detected via conformational recognition of HEL. This can be done in one of two ways: 1) FACS: use conjugate to bind CD169 macs and detect bound conjugate with anti-HEL Ab. The staining profile should look like that seen in Sup Figure 5A/B and confirmed as authentic in CL treated mice ie Sup Fig 5C. (where the staining should be abolished) 2) Confocal staining of CD169 macs using anti-CD169-HEL conjugate ie as was shown for anti-CD169-OVA in Sup Figure 1A.

The authors conclude that Ag targeted to CD169 Macs is presented to cognate B cells – perhaps this is conclusion is premature. I would like to see if B cell activation (down regulation of IgM and HEL binding is reduced in CL-treated mice). This would rule out non-specific effects HEL-conjugated mAb.

I understand there there are times when experiments only need to be performed once. However, I am concerned just how many experiments were only performed once. Below is a list of those experiments and my commentaries:

Fig 1C – I guess it's OK to do this experiment only once since it is not pivotal to the point of this manuscript and the data is black and white.

Fig 1D – I am not happy with this experiment having only been performed once: it is critical to the message of this paper and the results are far from clear (please see previous comment above).

Fig 3 A – I am OK with this experiment having been only performed once: it states the obvious and the results are black and white.

Fig 3D – ideally this experiment should be repeated but I acknowledge that it agrees with previous experiments.

Fig 4 Focuses on the idea that CD169 facilitates Ag retention and presentation to B cells. Here, mice were injected with 20-25 times more mAb than in the immunization experiments and the continued detection of

European Journal of Immunology

Ag on the cell surface is interpreted as evidence of Ag retention. If Ag retention and presentation to B cells via CD169 was critical, would this Ag retention not also be detected at the doses used to immunize mice?

Fig 4A – how many times was this done?
Fig 4F – I am not happy that this experiment was only performed once because it is integral to the message of this paper. Furthermore, I feel very uncomfortable being presented with highly processed data. Instead of showing the FACS plots (documenting down-regulation of HEL binding) we are given the data as ratios. I would like the raw data included as a supplementary figure and I would also like to see this experiment reproduced.

Minor typos:
Abstract: change: “specific APC forms…” to “specific APC is…”

I think that the following abbreviations have not been defined: SCN (Fig 2); FMO Fig 3E

The authors refers to “CD169 macs retaining intact Ag for several days”. This is incorrect. The authors show Ag retention for 2 days not several days. The definition of several means “3 or more”. As such their statement is misleading. Please reword.

Reviewer: 3

Comments to the Author
This is a straightforward study showing that targeting to CD169 with adjuvants can induce Ab responses. The difference between targeting CD169 vs. DEC205 qualitatively is not that dramatic, and previous studies have shown that one can induce immune responses by targeting CD169. Nevertheless, the results clearly are of interest to the field.

Fig. 1: The data overall are convincing and clear. In Fig. 1B, are the IgG1 and IgG2b Ab titers after targeting to DEC205 vs. CD169 significantly different or not? Evidence that the CLL treatment actually depleted macrophages needs to be provided, e.g., as supplementary data. Is the reduction in Ab titers by CLL treatment of DEC205 OVA targeted mice not statistically significant? This should be clarified.

Fig. 2. For Fig. 2C some of the GL7+ CD38+ B220+ cells in the spleen could be GC-independent memory B cell precursors. The authors could use PNA staining to differentiate between GC B cells and GC-independent memory B cell generation.

European Journal of Immunology

Fig. 3: For 3B, were the numbers of total CD4 T cells similar or only the frequency? Are the Ab titers significantly different after DEC205 vs. CD169 targeting or not?

Fig. 5: These data are missing a positive control, so it is hard to determine the significance of this level of Ab response. Why was LPS instead of poly I:C and anti-CD40 suddenly used in these experiments?

The non-scientific term ‘drastic’ is used, which means ‘acting rapidly or violently’ or ‘extreme in effect or action’. This term should be changed.

First revision – authors’ response – 21 October 2014

Reviewer 1:
The manuscript by Veninga et al. tackles an interesting and actual subject that is to target the CD169+ macrophage population, present in secondary lymphoid organs, for vaccination purposes. Previous work from several labs, including authors of this work, showed the particular abilities of these macrophages to capture antigen traveling in the lymph, and present it intact to B cells or even transfer it to other cells such as B cells or DC. In the present manuscript, the authors compared the T-dependent B cell immune responses of mice immunized with antigens targeted to CD169+ macrophages or to DC, using antigen conjugated to antibodies against CD169 or to DC205 (DC marker). They showed that the immune responses are stronger and long-lasting when the antigen is targeted to CD169+ macrophages than when it is targeted to DC. They described that CD169+ macrophages, in addition to retain antigen, are important for other events like the production of cytokines and enzymes crucial to promote B cell responses. The manuscript is well written, the experiments are well performed, and the conclusions are supported by the shown data and of relevance for immunologist community. I have however some major concerns that the authors should address.

1. The authors claim in the title and along the manuscript that these macrophages are “promoters of T cell dependent germinal centre B cell responses”. They used anti-CD40 antibody as adjuvant in practically the whole experimentation (except for Fig 5), which will bias towards a T cell dependent response already. I wonder what would happen in absence of anti-CD40 as part of the adjuvant, the CD169+ macrophages would promote a T cell dependent or T cell independent response at the spleen?? What about the comparison with the DC-targeted response in that case?? The authors should clarify this issue in the title and text, highlighting that the study is just a comparison between macrophage and DC abilities to induce a T-dependent humoral immune response when the antigen is targeted to one or the other cell type, or perform the needed experiments to answer the questions mentioned above.
We appreciate the valuable comments of reviewer 1. Our studies have indeed utilized anti-CD40 antibody

European Journal of Immunology

together with poly (I:C) as adjuvant in all experiments. While CD8 T cell responses do not depend on CD4 helper T cells when using poly(I:C) or anti-CD40 Ab as adjuvant (den Haan et al. JI 2007, 178:5429 and Bennett et al./Schoenberger et al./Ridge and Matzinger, Nature 1998), our experiments indicate that B cell responses after targeting to CD169+ macrophages and DEC205+ DCs still require the presence of CD4 helper T cells. In the absence of both poly(I:C) and anti-CD40, the B cell response is hardly detectable (Figure 1C). However, we have not determined the CD4 T cell dependency in the presence of only poly I:C as adjuvant. We agree with the notion that the main finding of our studies is the comparison between macrophage and DC abilities to induce a T cell dependent humoral immune response and have revised the title and manuscript text accordingly.

2. How does the antigen retention ability of CD169+ macrophages compare to that of DC at the times analyzed?

While we very clearly detect the presence of OVA in CD169+ macrophages at several time points (30 minutes – 48 hrs) after Ag targeting to CD169 by fluorescence microscopy using a polyclonal anti-OVA antibody, with this method we have only detected very few OVA-positive DCs only early (30 minutes-4 hrs) after DEC205 targeting. In contrast, DCs obtained from mice that were immunized with anti-DEC205:OVA are very well capable of inducing OT-I T cell proliferation in vitro, indicating that OVA targeted to DEC205+ DCs is efficiently being processed and presented. These observations suggest that CD169+ macrophages and DCs exhibit different rates of both Ag uptake as well as of Ag retention and/or processing.

Taking into account that a GC reaction takes 7 days more or less to form, and the role of antigen transfer from CD169+ macrophages to FDC (through B cells) for affinity maturation (Phan TG et al., Nat Immunol 2009), what about antigen retention at longer times than 48h at the surface of these cells?

The referee raises an interesting point and we have performed a new experiment to measure the presence of OVA at day 4 and day 7 after immunization. We detected low, but significant higher OVA staining after CD169 targeting when compared to control Ig targeting on day 4, but not day 7, after immunization when we measured extracellular OVA on non-fixed cells, which is more sensitive than measuring OVA in fixed cells. Since this preliminary experiment was performed with 3 mice per group, we did not include this result in the manuscript. However, our results suggest that CD169+ macrophages retain intact antigen for 2-4 days. Although the GC reaction takes 7 days to form, 2-4 days of intact extracellular antigen presentation could well be long enough to achieve antigen transport to the FDC network by follicular B cells as has been described by Phan et al.

European Journal of Immunology

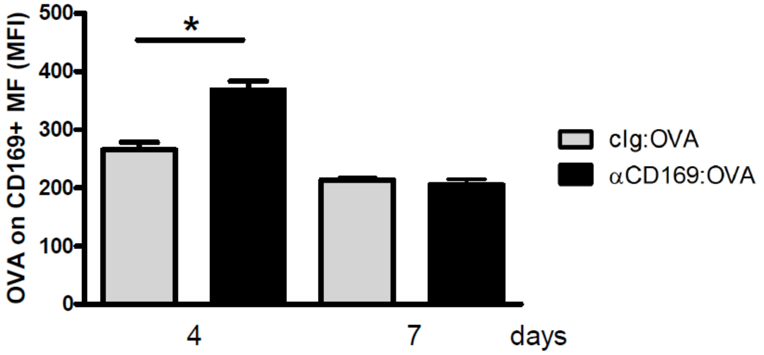

Figure R1: CD169+ Mj retain intact antigen for several days after immunization. Flow cytometric analysis of the retention of OVA on splenic CD169+ Mj 24 and 48 hrs after i.v. immunization with 20 mg of indicated mAb:OVA together with 25 mg aCD40 and 25 mg poly(I:C). Graph shows the geometric mean of the fluorescence intensity (mean ± SEM) of aOVA staining on CD169+ Mj gated as live, AF, CD169+ cells. Data is from a single experiment using three mice/group.

3. Is the upregulation of adhesion molecules and class II at macrophage surface similar when only adjuvant is administered to mice, in absence of OVA-conjugated anti-CD169 antibody?
The upregulation of adhesion molecules and class II on CD169+ macrophages is also observed when only adjuvant is administered to mice. We revised the text accordingly.

4. The elimination of macrophages with clodronate liposomes resulted in no production of the B cell stimulating factors IL-6, IL-27 and IFNa at the time analysed (2h) …….do the authors detect any sign of cytokine expression at later time points??.... is it just a matter of delayed kinetic or the spleen is not showing any sign of immune response at all (cytokine secretion, B cell activation, Ab production....)??
The cytokines shown in Figure 4E were analyzed at both 2 and 6 hr after adjuvant injection. At the 6 hr time point we only detected a significant increase in IFNa, but not IL-6 or IL-27, after immunization that was abrogated in clodronate liposome treated mice. This result indicates that type I IFN production is hampered for a longer period of time when macrophages in the marginal zone are depleted.
Although we do not detect Ab production or germinal center B cell responses after Ag targeting to CD169+ macrophages in clodronate liposome treated mice, we still detect Ab responses (Figure 1D), germinal center B cell responses (Figure 2C) and T cell responses (Figure S2C) when Ag is targeted to DEC205+ DCs in clodronate liposome treated mice. These results indicate that early production of B cell activating cytokines isdependent on the presence of macrophages in the marginal zone, but that B cell activation and Ab production is still feasible in the absence of macrophages.

Reviewer: 2
Comments to the Author

European Journal of Immunology

The manuscript by Veninga et al. dissects the role of CD169 macrophages in promoting CD4 T cell and B cell responses. The paper is methodical and well written. I only have minor comments and request the repetitions of key experiments. If the repetition of these experiments is not feasible, I would like the authors to highlight in the discussion that their conclusions are based on “one-off” experiments (and are indicative rather than conclusive).

Figure 1A shows OVA-specific responses in mice injected with 1 ug Ig, a-CD169 or a-DEC205 in the presence of 25 ug aCD40 + 25 ug polyIC. On day 9, naïve and cIg immunized mice have an 1/1000 titer. By contrast, aDEC or aCD169 targeted mice have 1/5000 or 1/6000 titers. Thus targeted mice have 5-6 times higher responses. In Figure 1C there appears to be approximately 10-fold difference between targeted and non-targeted. However, in Figure 1D, in mice immunized under the same conditions, there is only a 2-fold difference between targeted delivery and non-targeted delivery: the cIg-OVA: OD 0.5 and aDEC or a-CD169 give an OD 1.00. It appears that targeting is barely advantageous in this experiment only providing a 2-fold enhancement. If that is the case maybe it was not the best experiment to deplete macrophages in, since targeting did not seem to work well.

We thank reviewer 2 for the raised comments. It is indeed true that targeting with aDEC205 or aCD169 in Figure 1A resulted in 5-6 times and in Figure 1C 9 times stronger responses when compared to cIg targeting. These figures depict the serum titers that result in OD450 higher than 0.1. In the original Figure 1D only a 2-fold difference was observed between targeted versus non-targeted Ag delivery when the OD of one fixed serum dilution was measured. However, since the OD of a certain serum dilution cannot be compared to serum titers, conclusions about targeting efficiency could not be drawn from the original figure 1D.

Since the necessity of reproducing the experiment shown in Figure 1D was also raised (see below), we repeated this experiment in which we treated mice with clodronate liposomes 8 days before immunization (6 mice per group). The 8 day time period allowed for the reappearance of most phagocytic cells including DC subsets in the spleen but macrophages in the marginal zone were still depleted (Fig. S2A-B). Similar results were obtained in this new experiment and the results are shown in new Figure 1D. In this new figure 1D we show the serum titers with OD450 > 0.1 as we do in figures 1A-C, instead of the OD of a fixed serum dilution. These new data demonstrate that targeting with aDEC205 or aCD169 induces 8-10 times higher responses than non-targeted immunization, which is in the same order of magnitude as the responses shown in Figure 1A and C.

Figure 4F – what are the units of the y-axis; Fig. 4A and C lack legends.

The unit of the y-axis of the old figure 4F was the ratio of the geometric mean fluorescence intensity of the indicated staining over the background staining (FMO, fluorescence minus one), but because of the remarks of reviewers 2 and 3 about this representation of the data we have adjusted this figure. Figure 4F now shows the geometric mean fluorescence intensity of the indicated markers HEL and IgM.

Furthermore, legends showing which lines represent which groups are included in Figures 4A and C and in Figure 4C we included the units of the y-axis.

The authors have conjugated HEL to mAb. Does this conjugate retain the ability to recognize CD169 and is the conjugated HEL still recognized by MD4 transgenic? I would like to see data that the conjugate bind its target and that this binding was detected via conformational recognition of HEL. This can be done in one of two ways:
1) FACS: use conjugate to bind CD169 macs and detect bound conjugate with anti-HEL Ab. The staining profile should look like that seen in Sup Figure 5A/B and confirmed as authentic in CL treated mice ie Sup Fig 5C. (where the staining should be abolished) 2) Confocal staining of CD169 macs using anti-CD169-HEL conjugate ie as was shown for anti-CD169-OVA in Sup Figure 1A.
We have performed several control experiments to ensure that the HEL:mAb conjugates retain their binding capacity to CD169. First, we performed immunofluoroscence stainings on spleen sections with either anti-CD169 or anti-CD169:HEL. Binding was detected with an anti-rat IgG or with a polyclonal rabbit anti-HEL and subsequently an anti-rabbit Ig (Figure S8A). Staining of anti-CD169:HEL with either an anti-rat IgG or with anti-HEL and anti-rabbit Ig was equally good as staining with anti-CD169 together with anti-rat IgG. Next, we performed an ELISA to evaluate the mAb:HEL conjugates for the presence of HEL. We coated mAb:HEL conjugates on Maxisorb plates in serial dilutions and detected the presence of HEL with a polyclonal anti-HEL antibody (Figure S8B). This serial dilution graph shows similar amounts of HEL are coupled to the different antibodies used in this study. Finally, we performed an in vivo targeting experiment to show that anti- CD169:HEL targets to CD169+ macrophages in the spleen. Mice were injected i.v. with either 1 or 20 mg cIg:HEL or anti-CD169:HEL. Spleens were collected after 30 min and an anti-CD169 and anti-HEL staining were performed on single cell suspensions. Figure S8C shows that both 1 and 20 mg anti-CD169:HEL can be detected on the cell surface of splenic CD169+ macrophages.

The authors conclude that Ag targeted to CD169 Macs is presented to cognate B cells – perhaps this is conclusion is premature. I would like to see if B cell activation (down regulation of IgM and HEL binding is reduced in CLtreated mice). This would rule out non-specific effects HEL-conjugated mAb.
We agree with the reviewer that a control for non-specific targeting is essential to be able to evaluate the actual targeting efficiency. It is, however, our opinion that non-specific effects of HEL-conjugated mAb can also be determined by the use of the cIg:HEL immunization. This is a non-targeted Ab isotype control antibody:HEL conjugate. We analyzed the IgM downregulation and reduction of HEL binding on HEL-specific B cells after immunization with anti-CD169:HEL and compared this immunization with non-specific cIg:HEL.

I understand there are times when experiments only need to be performed once. However, I am concerned just how many experiments were only performed once. Below is a list of those experiments and my commentaries:

European Journal of Immunology

We agree with the reviewer that several experiments needed to be repeated and we complied with these wishes as much as we were able to. However, our ethical committee felt it was ethically unjustified to repeat experiments that are in agreement with other experiments. For this reason we were not able to repeat the experiment shown in Figure 1C, 3A and 3D. Fig 1C – I guess it's OK to do this experiment only once since it is not pivotal to the point of this manuscript and the data is black and white.

Fig 1D – I am not happy with this experiment having only been performed once: it is critical to the message of this paper and the results are far from clear (please see previous comment above).
We have repeated the experiment using six mice per group and observed similar results in both experiments. The data of these experiments are shown in Figure 1D and Figure S2.

Fig 3 A – I am OK with this experiment having been only performed once: it states the obvious and the results are black and white.
Fig 3D – ideally this experiment should be repeated but I acknowledge that it agrees with previous experiments.
Fig 4 Focuses on the idea that CD169 facilitates Ag retention and presentation to B cells. Here, mice were injected with 20-25 times more mAb than in the immunization experiments and the continued detection of Ag on the cell surface is interpreted as evidence of Ag retention. If Ag retention and presentation to B cells via CD169 was critical, would this Ag retention not also be detected at the doses used to immunize mice?
We have performed two independent experiments to address the presence of OVA on CD169+ macrophages after targeting with 1 ug CD169:OVA (Figure R2A-B). Figure R1A shows that 4 hr after targeting with 1 mg of aCD169:OVA OVA can be detected on CD169+ macrophages. Figure R1B shows that when we compared 1 and 20 mg mAb:OVA targeting side by side, the level of OVA is lower after 1 mg aCD169:OVA targeting compared to 20 mg aCD169:OVA, while 1-20 ug cIg:OVA background levels are similar and not different from the background of OVA staining in naïve mice. These data show that Ag retention can also be detected at the dosis used to immunize mice.

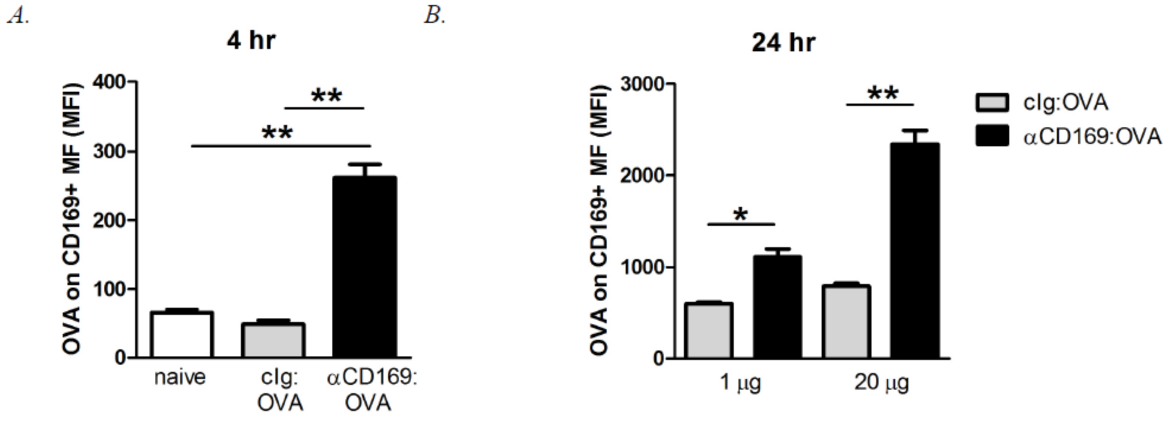

Figure R2. OVA can be detected on CD169+ Mj after targeting with 1 ug CD169:OVA. (A) Flow cytometric analysis of the presence of OVA on splenic CD169+ Mj 4 (A) and 24 (B) hrs after i.v. immunization with 1 mg (A) and 1 or 20 mg (B) of indicated mAb:OVA together with 25 mg aCD40 and 25

European Journal of Immunology

mg poly(I:C). Graphs shows the geometric mean of the fluorescent intensity (mean ± SEM) of aOVA staining on CD169+ Mj gated as live, AF, CD169+ cells. Data in both graphs is from a single experiment using three mice/group. Data in (A) were analyzed by ANOVA with Bonferroni's correction. Pvalue indicator ** refers to P < 0.005. Data in (B) were analyzed by a two-tailed student-T-test. P-value indicator *, and ** refers to P < 0.05, and P < 0.005 respectively.

Fig 4A – how many times was this done?

Day 1 (24 hr) has been performed 3 times, and day 2 (48 hr) twice. The text is revised accordingly.

Fig 4F – I am not happy that this experiment was only performed once because it is integral to the message of this paper. Furthermore, I feel very uncomfortable being presented with highly processed data. Instead of showing the FACS plots (documenting down-regulation of HEL binding) we are given the data as ratios. I would like the raw data included as a supplementary figure and I would also like to see this experiment reproduced.

We have repeated this experiment as requested. In this new experiment we detected a similar, but not significant, decrease in IgM and HEL binding after targeting to CD169+ macrophages (Figure R3). As the reviewer states in the beginning of the review, these results are indicative rather than conclusive and we have revised the text accordingly. As requested, raw data and gating strategies are provided in supplementary figure S8.

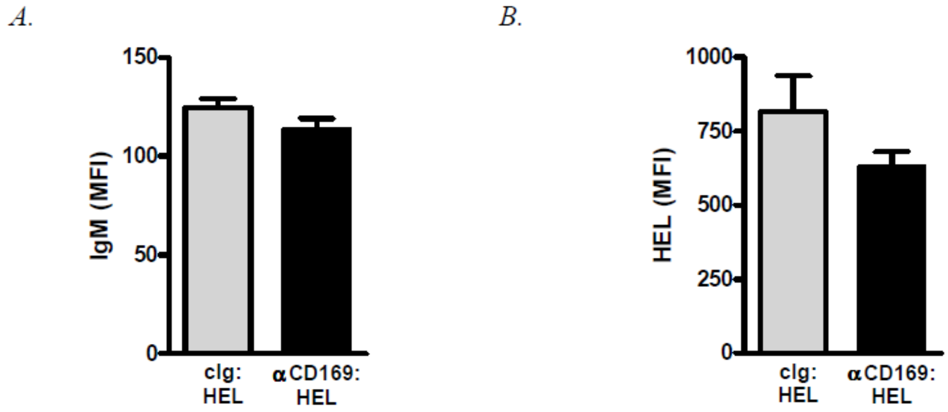

Figure R3: IgM surface expression and capacity to bind HEL of HEL-specific MD4 B cells after antigen targeting to CD169+ Mj. B6 mice received CFSE labeled MD4 transgenic cells and immunized with 1 mg mAb:HEL conjugate together with 25 mg aCD40 and poly(I:C). MD4 tg B cells (live/dead marker-/ B220+/ CFSE+) were analyzed for cell surface IgM expression (A) and HEL binding (B) 24 hrs after immunization. Data represents the geometric mean of the fluorescent intensity ± SEM of the relevant marker of a single experiment with five to six mice/group. Gating strategy is shown in Fig. S8. B220+, CFSE+ B cells were checked for the presence of IgDa+ in a separate staining to confirm that these cells are MD4 tg B cells. Data were analyzed by ANOVA with Bonferroni's correction.

European Journal of Immunology

Minor typos:

Abstract: change: “specific APC forms…” to “specific APC is…”

Text is revised accordingly.

I think that the following abbreviations have not been defined: SCN (Fig 2); FMO Fig 3E

Text is revised accordingly

The authors refer to “CD169 macs retaining intact Ag for several days”. This is incorrect. The authors show Ag retention for 2 days not several days. The definition of several means “3 or more”. As such their statement is misleading. Please reword.

The text is revised to “at least two days”

Reviewer: 3

Comments to the Author

This is a straightforward study showing that targeting to CD169 with adjuvants can induce Ab responses. The difference between targeting CD169 vs. DEC205 qualitatively is not that dramatic, and previous studies have shown that one can induce immune responses by targeting CD169. Nevertheless, the results clearly are of interest to the field.

Fig. 1: The data overall are convincing and clear. In Fig. 1B, are the IgG1 and IgG2b Ab titers after targeting to DEC205 vs. CD169 significantly different or not?

The data were analyzed by a Kruskal-Wallis test with Bonferroni’s correction, $p > 0.0167$ (comparison with 3 groups; cIg:OVA, DEC205:OVA and CD169:OVA). With this analysis IgG1 and IgG2b Ab titers after targeting to DEC205 when compared to CD169 were not significantly different.

Evidence that the CLL treatment actually depleted macrophages needs to be provided, e.g., as supplementary data.

We used clodronate liposome treatment to specifically deplete macrophages from the marginal zone. To achieve this we treated the mice with clodronate liposomes 7-8 days before immunization. This time period allows phagocytic cells such as DCs to repopulate the spleen again, with the exception of macrophages in the marginal zone which take longer to recover (van Rooijen et al. J. Leuk. Biol. 1989, 45: 97). Data showing that macrophages in the marginal zone are indeed depleted at the day of immunization are provided in Figure S2A. In contrast, DC subsets have returned in the spleen at the day of immunization (Figure S2B). We further show that the induction of OVA-specific IFNg producing CD8+ and CD4+ T cells is only affected in clodronate liposome treated mice when OVA is targeted to CD169+ macrophages and not after DEC205 targeting (Figure S2C). This further indicates that the clodronate liposome treatment did not hamper DC activity, but specifically affects macrophages in the marginal zone.

European Journal of Immunology

Is the reduction in Ab titers by CLL treatment of DEC205 OVA targeted mice not statistically significant? This should be clarified.

Antibody titers between DEC205:OVA targeting in B6 vs CL treated mice are not significantly different in both experiments that are performed; the p-value when using a Kruskal-Wallis test with Bonferroni's correction > 0.025 (comparing only 2 groups; DEC205 in B6 versus CL treated mice).

Fig. 2. For Fig. 2C some of the GL7+ CD38+ B220+ cells in the spleen could be GC-independent memory B cell precursors. The authors could use PNA staining to differentiate between GC B cells and GC-independent memory B cell generation.

The reviewer raises a justified concern in that GL7+CD38+B220+ B cells could include GC independent memory B cell precursors. We initially gated on GL7+CD38low-neg B220+ cells and we have adjusted the gating strategy according to recent literature (Taylor et al. J. Exp. Med. 2012, 209: 597); the OVA binding B cells that are plotted in Figure 2B-C are GL7 positive and CD38 negative. B cells with these characteristics are thought to be true GC B cells and by using this gating strategy we excluded GC-independent memory B cell precursors that are CD38pos. Gates were set on the appropriate FMO's and the gating strategy is included in the supplementary data (Figure S3).

Fig. 3: For 3B, were the numbers of total CD4 T cells similar or only the frequency?

Both the frequency as well as the total number of Ag specific CD4 T cells were similar.

Are the Ab titers significantly different after DEC205 vs. CD169 targeting or not?

The Ab titers were not significantly different between DEC205 and CD169 targeting.

Fig. 5: These data are missing a positive control, so it is hard to determine the significance of this level of Ab response. Why was LPS instead of poly I:C and anti-CD40 suddenly used in these experiments?

The purpose of this experiment is to show that targeting of Ag to CD169+ macrophages utilizing a different targeting strategy (without using Ab) also gives rise to antibody production. Using this Ag targeting strategy we were able to rule out Fc-receptor involvement and to show that a different adjuvant such as LPS together with CD169 targeting can also induce B cell responses. We agree that it is hard to determine the Ab response quantitatively, but this experiment does indicate that Ag bound to CD169 stimulate B cell responses.

The non-scientific term 'drastic' is used, which means 'acting rapidly or violently' or 'extreme in effect or action'. This term should be changed.

The term "drastic" has been replaced with "significantly".

Peer review correspondence

Second Editorial Decision – 18 November 2014

Dear Dr. den Haan,

It is a pleasure to provisionally accept your manuscript entitled "Antigen targeting reveals splenic CD169+ macrophages as promoters of germinal center B cell responses" for publication in the European Journal of Immunology. For final acceptance, please follow the instructions below and return the requested items as soon as possible as we cannot process your manuscript further until all items listed below are dealt with.

Please note that EJI articles are now published online a few days after final acceptance (see Accepted Articles: http://onlinelibrary.wiley.com/journal/10.1002/(ISSN)1521-4141/accepted). The files used for the Accepted Articles are the final files and information supplied by you in Manuscript Central. You should therefore check that all the information (including author names) is correct as changes will NOT be permitted until the proofs stage.

We look forward to hearing from you and thank you for submitting your manuscript to the European Journal of Immunology.

Yours sincerely,
Katharina Schmidt

on behalf of Dr. Steffen Jung

Dr. Katharina Schmidt
Editorial Office
European Journal of Immunology
e-mail: ejied@wiley.com
www.eji-journal.eu
